# The IMiDs targets IKZF-1/3 and IRF4 as novel negative regulators of NK cell-activating ligands expression in multiple myeloma

**DOI:** 10.18632/oncotarget.4603

**Published:** 2015-06-23

**Authors:** Cinzia Fionda, Maria Pia Abruzzese, Alessandra Zingoni, Francesca Cecere, Elisabetta Vulpis, Giovanna Peruzzi, Alessandra Soriani, Rosa Molfetta, Rossella Paolini, Maria Rosaria Ricciardi, Maria Teresa Petrucci, Angela Santoni, Marco Cippitelli

**Affiliations:** ^1^ Department of Molecular Medicine, Istituto Pasteur-Fondazione Cenci Bolognetti, Sapienza University of Rome, Rome, Italy; ^2^ Istituto Italiano di Tecnologia, CLNS@Sapienza, Sapienza University of Rome, Rome, Italy; ^3^ Division of Hematology, Department of Cellular Biotechnologies and Hematology, Sapienza University of Rome, Rome, Italy; ^4^ Istituto Mediterraneo di Neuroscienze Neuromed, Pozzilli, Italy

**Keywords:** IMiDs, multiple myeloma, natural killer, NKG2DLs, DNAM-1Ls

## Abstract

Immunomodulatory drugs (IMiDs) have potent anti-tumor activities in multiple myeloma (MM) and are able to enhance the cytotoxic function of natural killer (NK) cells, important effectors of the immune response against MM. Here, we show that these drugs can enhance the expression of the NKG2D and DNAM-1 activating receptor ligands MICA and PVR/CD155 in human MM cell lines and primary malignant plasma cells. Depletion of cereblon (CRBN) by shRNA interference strongly impaired upregulation of these ligands and, more interestingly, IMiDs/CRBN-mediated downregulation of the transcription factors Ikaros (IKZF1), Aiolos (IKZF3) and IRF4 was critical for these regulatory mechanisms. Indeed, shRNA knockdown of IKZF1 or IKZF3 expression was both necessary and sufficient for the upregulation of MICA and PVR/CD155 expression, suggesting that these transcription factors can repress these genes; accordingly, the direct interaction and the negative role of IKZF1 and IKZF3 proteins on MICA and PVR/CD155 promoters were demonstrated. Finally, MICA expression was enhanced in IRF4-silenced cells, indicating a specific suppressive role of this transcription factor on MICA gene expression in MM cells.

Taken together, these findings describe novel molecular pathways involved in the regulation of MICA and PVR/CD155 gene expression and identify the transcription factors IKZF-1/IKZF-3 and IRF4 as repressors of these genes in MM cells.

## INTRODUCTION

Multiple myeloma (MM) is an incurable hematologic cancer characterized by clonal expansion of cancerous plasma cells in the bone marrow [1, 2] and its development is supported by a progressive impairment of immunosurveillance, mainly attributable to T and NK cell alterations [3-5]. NK cells significantly contribute to anti-tumor immune response, due to their ability to recognize and lyse cancer cells. The NK cell cytotoxic activity is controlled by integrated signals delivered from a complex set of inhibitory and activating receptors [6, 7]. In this regard, NKG2D and DNAM-1 are two activating receptors shown to play a prominent role in tumor immunosurveillance.

NKG2D interacts with multiple ligands, including MHC class I-related chain A/B (MICA/B) and UL16 binding proteins (ULBPs), while DNAM-1 recognizes two nectin-like proteins, the poliovirus receptor (PVR/CD155) and Nectin-2 (Nec-2).

NKG2D ligands (NKG2DLs) show a restricted expression on healthy cells, however they are frequently overexpressed in a wide range of pathological conditions, including cancer and infection. In tumor cells, their expression is controlled at different levels by complex mechanisms only partially characterized. In particular, oncogenic proliferative and stress signaling pathways linked to the tumorigenic process, such as DNA damage response (DDR), can increase their basal expression [8-10]. Moreover, to escape immune cell mediated recognition, tumors can downregulate NKG2DLs expression, via proteolytic shedding from the cell surface and/or excretion in exosomes [11, 12].

DNAM-1 ligands (DNAM1Ls) are ubiquitously expressed in various tissues, and their levels increase in different tumor cells, but the mechanisms leading to their regulation are largely unknown [13]. In this regard, evidence is accumulating that the engagement of NKG2D and DNAM-1 activating receptors is critical for NK-mediated killing of myeloma cells, which express NKG2D and DNAM-1 ligands [4, 14-18]. Thus, improving NK cell responsiveness may be a promising therapeutic approach to treat MM; in particular, the modulation of the balance between activating and inhibitory NK cell signals and sensitization of cancer cells to NK cell mediated-cytotoxicity may significantly contribute to enhance anti-myeloma immune response.

Of note, among novel therapeutic agents used in MM clinical management, IMiDs [thalidomide, lenalidomide (CC-5013) and pomalidomide (CC4047)], have the ability to modulate humoral as well as cellular components of the innate and adaptive immune responses, including NK cell activity against cancer cells [5, 19-22]. In particular, an increased number of NK cells expressing higher levels of the activating receptors NKG2D, NKp30 and NKp44 has been observed in MM patients during IMiDs therapy [23, 24]; moreover, IMiDs enhance NK cell antibody-dependent cell-mediated cytotoxicity (ADCC) activity toward MM cells [25], and can sensitize myeloma cells to NK cell cytotoxicity by inhibiting the expression of PD-L1 [26].

In the last few years, the mechanism of action of IMiDs has becoming increasingly clear. The cellular target of these drugs is cereblon (CRBN), a ubiquitous protein which functions as a substrate receptor for the Cullin-4-RING Ubiquitin Ligase (CLR4) complex, containing Cullin-4 (CUL4), DNA damage binding protein-1 (DDB1) and the RING-finger protein (ROC1) [29, 30].

CRBN is essential for the anti-myeloma activity of IMiDs and loss of this protein causes drug resistance in MM cells [30, 31]. IMiDs binding to CRBN alters the function of the CLR4 [29]; in particular enforced binding of IMiDs to CRBN is able to disrupt the recruitment of some substrates and, at the same time, promotes the ubiquitination of other proteins, suggesting the possibility that a neomorphic structure is involved [32, 33].

In this context, recent papers described the capability of IMiDs to induce CRBN-dependent degradation of the Ikaros family zinc finger protein-1 (IKZF1, Ikaros) and 3 (IKZF3, Aiolos) [34, 35], two transcription factors involved in lymphoid development and differentiation and highly expressed in B cell malignancies, including MM [36, 37]. Loss of these proteins was shown to mediate important therapeutic activities of IMiDs, such as increased IL-2 production by T cells [35, 38] or anti-proliferative effects in MM cells [34, 35]. Interestingly, in MM cells IKZF1 and IKZF3 have also emerged as positive regulators of the expression of the IRF4 gene [34-36], a haemopoietic cell-restricted transcription factor, identified as a key regulator of malignancy-specific gene expression in MM [39, 40]. In this regard, IMiDs were found to inhibit IRF4 expression at transcriptional level, mainly via downregulation of IKZF1/3 transcription factors [34].

In this study, we define novel regulatory mechanisms of NK cell-activating ligand gene expression in MM cells. We demonstrate that IMiDs can upregulate MICA and PVR/CD155 surface expression, enhancing NK cell recognition and killing. A prominent role in these regulatory mechanisms is played by the transcription factors IKZF1/3 and IRF4, able to repress the basal transcription of *mica* and *pvr* gene expression. Lenalidomide-induced downregulation of these transcription factors leads to de-repression of *mica* and *pvr* promoter activity, and consequently to increased gene transcription.

Thus, we identified IKZF1/3 and IRF4 as “druggable” transcriptional repressors of NK cell-activating ligand expression in MM cells.

## RESULTS

### IMiDs upregulate MICA and PVR/CD155 expression on human multiple myeloma cells and enhance their recognition by NK cells

**Figure 1:**
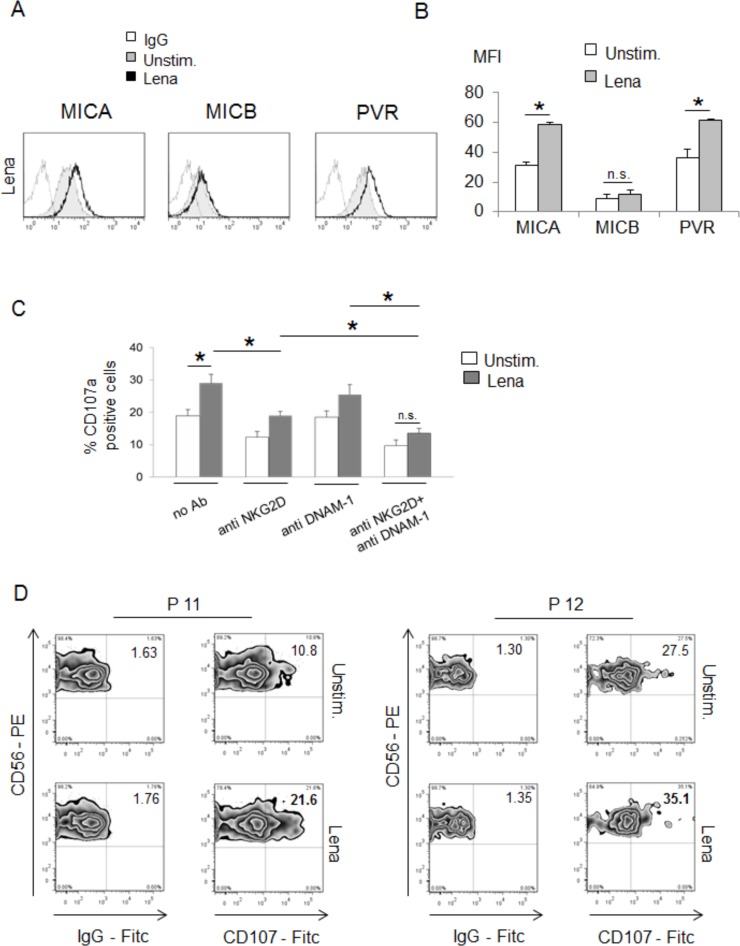
IMiDs upregulate MICA and PVR/CD155 expression on human Multiple myeloma cells and enhance their recognition by NK cells **A.** MICA, MICB and PVR/CD155 surface expression were analyzed by flow cytometry on SKO-007(J3) cells treated with lenalidomide (Lena) (10 μM) for 72h. The grey colored histograms represent basal expression of the indicated ligand, while thick black histograms represent the expression after treatment with the drug. Data are representative of one out of four independent experiments. **B.** The MFI of MICA, MICB and PVR/CD155 were calculated based on at least four independent experiments and evaluated by paired Student *t* test (*P < 0.05). Histograms represent the MFI of specific mAb - MFI of isotype control. **C.** NK cells, prepared from PBMCs of healthy donors, were incubated with SKO-007(J3) cells, untreated or treated with lenalidomide (Lena) for 72h, and used as target cells in a degranulation assay. The assay was performed at the effector:target (E:T) ratio of 2.5:1. After 3 hours at 37°C, cells were stained with anti-CD56, anti-CD3 and anti-CD107a mAbs. Cell surface expression of CD107a was analyzed on FSC/SSC-gated and CD56^+^CD3^−^ cells. In order to evaluate the role of NKG2D and DNAM-1, the assay was performed in parallel treating NK cells with blocking anti-DNAM-1 or anti-NKG2D antibodies. The MFI of CD107a were calculated based on at least three independent experiments and evaluated by paired Student *t* test (*P < 0.05). For each treatment, histograms represent the MFI of specific mAb - MFI of isotype control. **D.** CD138^−^ bone marrow cells, cultured for 2 days in complete medium supplemented with IL-2 (200 U/mL), were incubated with purified autologous myeloma cells, untreated or treated with lenalidomide (Lena) for 48h, and used as target cells in a degranulation assay. The assay was performed at the effector:target (E:T) ratio of 2.5:1. After 3 hours at 37°C, cells were stained with anti-CD56, anti-CD3 and anti-CD107a mAbs. Cell surface expression of CD107a was analyzed on CD56^+^CD3^−^ cells. Results obtained from two patients (P11 and P12) are represented.

In the last few years, our laboratory has investigated the expression and regulation of different NKG2D and DNAM-1 ligands on human MM cells in response to anti-myeloma agents [16, 27, 41]. In this context, we and other authors have initially reported the capability of lenalidomide to increase the expression of several NK cell-activating ligands on MM cells [27, 28]; however, the molecular mechanisms involved have not been investigated yet. To better analyse the effects of IMiDs on the expression of NK cell-activating ligands, we initially performed flow cytometric analyses on SKO-007(J3) cells, a MM cell line known to basally express MICA/B and PVR/CD155 [16], after 72h-treatment with micromolar concentrations of lenalidomide or pomalidomide. We observed that these drugs upregulate the basal expression of MICA and PVR/CD155 on SKO-007(J3) cells, with no significant effects on MICB levels (Fig. 1A and 1B and Suppl. Fig. 1A and 1B). Similar data were also obtained in other MM cell lines that constitutively express either one of these ligands: ARP-1 and JJN3 cells for MICA and KMS27 and OPM-2 cells for PVR/CD155 (Suppl. Fig. 2). Moreover, where not expressed, we did not observe a neo-induction of these ligands in IMiDs-treated cells (data not shown).

We could confirm these results also in CD138^+^ MM cells from the bone marrow of MM patients, showing higher surface levels of MICA and/or PVR/CD155 following treatment with lenalidomide (Table 1 and 2). Of note, in some patient-derived PCs, the drug did not show a significant effect on either MICA or PVR/CD155, independently from the clinical stage of disease and from basal level expression of these ligands, suggesting that different gene-specific mechanisms of regulation could be involved.

**Table 1:** Clinical parameters of MM patients

Patient no.	Sex/Age	Clinical Stage	Monoclonal Ig	% PCs in BM
1	M/67	Relapse	IgG-k	37
2	F/78	Relapse	IgG-k	11
3	M/58	Relapse	IgG-k	49
4	F/73	Smoldering	IgG-k	38
5	F/79	Onset	IgG-k	65
6	M/79	Smoldering	IgG-L	32
7	F/73	Relapse	IgG-k	19
8	F/58	Relapse	IgG-k	28
9	M/61	Relapse	Micro-k	60
10	F/73	Smoldering	IgA-L	34
11	F/74	Onset	IgG-k	22
12	M/74	Onset	IgG-k	22
13	F/74	Onset	IgG-L	72
14	F/61	Relapse	IgG-k	39
15	M/79	Relapse	IgG-k	43

Patients were classified according to Durie and Salmon's Staging System.

**Table 2:** Upregulation of MICA and PVR/CD155 expression on patient-derived PCs cells upon treatment with lenalidomide

Patient	MICA		MICB		PVR/CD155	
	Unstim.	Lena	Unstim.	Lena	Unstim.	Lena
1	1.7	1.7	1.5	1.5	4.9	13.9
2	2.4	1	2.1	2.4	1	6
3	2.4	4.9	2.5	1	2.3	13.9
4	5.9	8.1	1.3	1.2	1.3	1.3
5	1.7	4.6	1	1.7	3.7	3
6	1.6	1	1	1.2	1	14.4
7	1	1	1,4	1.4	5.5	73.6
8	13	19.3	1.7	2	1.2	1
9	1	2.6	1	1.4	2.2	1.7
10	2.3	3.8	1	1.6	1.3	4.3

MICA, MICB and PVR/CD155 surface expression was analyzed by flow cytometry on patient MM cells treated with lenalidomide (Lena) for 48h. For each treatment, value ratio between MFI of specific mAb and MFI of isotype control was reported.

As regards the other NKG2D and DNAM-1 ligands, SKO-007(J3) cells express low or undetectable levels of ULBP2/5/6 or ULBP1, ULBP3 and Nec-2 respectively, and lenalidomide did not modify their surface levels (Suppl. Fig. 3). These treatments did not affect the cell viability of these cell lines after 72h-treatment, as assessed by PI staining (data not shown).

To evaluate the functional consequence of IMiDs-induced changes of MICA and PVR/CD155 expression, we analyzed the lysosomal marker CD107a (a surrogate marker for granule mobilization [42]) on NK cells isolated from healthy donors against SKO-007(J3) cells, untreated or treated with lenalidomide as described above, by FACS analysis. As shown in Fig. 1C, basal expression of CD107a on NK cells was enhanced when co-cultured with SKO-007(J3) target cells exposed to lenalidomide; this effect was significantly inhibited by the combined blocking anti-NKG2D plus anti-DNAM-1 mAbs, indicating that stimulation of NK cell degranulation was dependent on both NKG2D and DNAM-1 activation. Accordingly, a higher capability of degranulation was also observed in patient-derived NK cells against lenalidomide-treated autologous MM targets (Fig. 1D).

Altogether, these data show that IMiDs treatment of human MM cells enhances MICA and PVR/CD155 membrane expression by increasing their susceptibility to NK cell recognition and killing.

### MICA and PVR/CD155 upregulation by IMiDs involves transcriptional mechanisms

**Figure 2:**
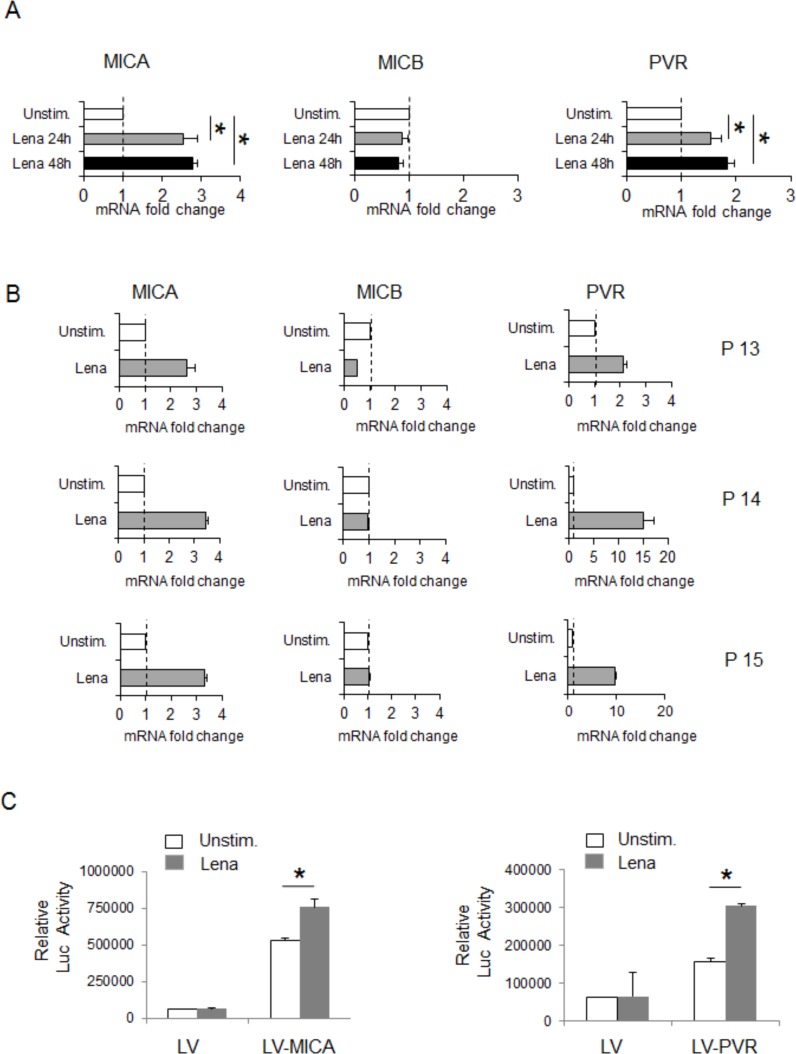
Lenalidomide increases MICA and PVR/CD155 mRNA expression and promoter activation in SKO-007(J3) cells **A.** Real Time PCR analysis of total mRNA obtained from SKO-007(J3) cells, untreated or treated with lenalidomide (Lena) as described above for 24h and 48h. Data, expressed as fold change units, were normalized with GAPDH and referred to the untreated cells, considered as calibrator and represent the mean of 3 experiments (*P < 0.05). **B.** Real Time PCR analysis of total mRNA obtained from purified CD138^+^cells untreated or treated with lenalidomide (Lena) for 24h in complete medium supplemented with 20 ng/ml IL-3 and 2 ng/ml IL-6. Data, expressed as fold change units, were normalized with GAPDH and referred to the untreated cells considered as calibrator. **C.** SKO-007(J3) cells were infected with lentivirus encoding LV control, LV-MICA or LV-PVR/CD155 promoter, obtained as described in Materials and Methods. After 48h treatment with lenalidomide (Lena), cellular medium were harvested and analyzed for the Gaussia Luciferase and SEAP assays. Results are expressed as relative luciferase activity normalized to SEAP activity and represent the mean value from four independent experiments (*P < 0.05).

To examine whether the upregulation of MICA and PVR/CD155 membrane expression by IMiDs could be associated with increased mRNA levels, total RNA was isolated from SKO-007(J3) cells exposed to lenalidomide or pomalidomide and analyzed by Real-Time qRT-PCR. As shown in Fig. 2A (and Suppl. Fig. 4), we found a significant increase of MICA and PVR/CD155 mRNA levels in treated cells, whereas, in accordance with the results at protein level, we did not detect any changes of MICB transcripts. Moreover, similar results were obtained in lenalidomide-treated MM cells isolated from three different patients (Fig. 2B).

To explore the possibility that these drugs could increase MICA and PVR/CD155 expression at transcriptional level, we analyzed their effect on SKO-007(J3) cells stably infected with a lentivirus carrying a MICA or a PVR/CD155 5′-flank (promoter fragment) upstream of a GLuc reporter gene. As shown in Fig. 2C, lenalidomide enhanced the activity of the reporter gene driven by a 1.2 kb fragment of the *mica* or a 1.3 kb fragment of the *pvr* gene promoter.

Thus, MICA and PVR/CD155 mRNA expression and promoter activity are enhanced by IMiDs in MM cells.

### IMiDs-induced MICA and PVR/CD155 upregulation is CRBN-dependent and involves IKZF1/3 transcription factors

**Figure 3:**
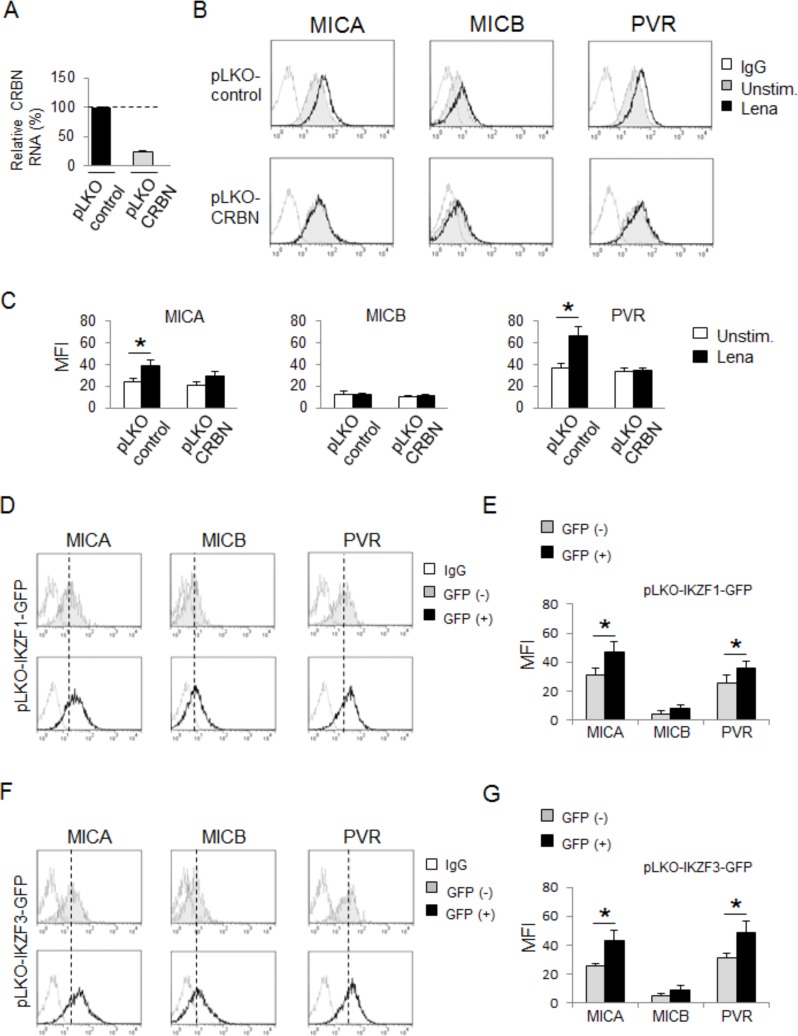
MICA and PVR/CD155 upregulation by IMiDs is CRBN-dependent and involves IKZF1/3 transcription factors **A.** Total mRNA was obtained from SKO-007(J3) cells transiently infected with lentivirus pLKO-shRNA-CRBN or pLKO non-targeting shRNA (control) (72h) and analyzed for CRBN mRNA expression by Real-Time qRT-PCR. Data were normalized with GAPDH and referred to the cells infected with non-target shRNA, considered as calibrator. **B.** MICA, MICB and PVR/CD155 surface expression were analyzed by flow cytometry on SKO-007(J3) pLKO non-target shRNA or pLKO-shRNA-CRBN cells, treated with lenalidomide (Lena) as described above. The grey colored histograms represent basal expression of the indicated ligand, while thick black histograms represent the expression after treatment with the drug. Data are representative of one out of five independent experiments. **C.** The MFI of MICA, MICB and PVR/CD155 were calculated based on at least five independent experiments and evaluated by paired Student *t* test (*P < 0.05). For each treatment, histograms represent the MFI of specific mAb - MFI of isotype control. **D.**, **F.** MICA, MICB and PVR/CD155 surface expression were analyzed by flow cytometry on pLKO-IKZF1/GFP or **F.**, **G.**) pLKO-IKZF3/GFP -lentivirus transiently infected SKO-007(J3) cells (72h), by gating on GFP^+^ and GFP^−^ cells as indicated in Suppl. Fig. 7C. Data are representative of one out of four independent experiments. The grey colored histograms represent the expression of the indicated ligand in GFP^−^ cells while thick black histograms represent the expression of the indicated ligand in GFP^+^ infected cells. **E.**, **G.** The MFI of MICA, MICB and PVR/CD155 were calculated based on at least four independent experiments and evaluated by paired Student *t* test (*P < 0.05). For each treatment, histograms represent the MFI of specific mAb - MFI of isotype control.

To investigate the role of CRBN in MICA and PVR/CD155 upregulation by IMiDs, we first treated SKO-007(J3) cells with phthalimide, a thalidomide analogue lacking the glutarimide moiety, the structural component mediating binding to CRBN [32]. We did not observe any changes in ligand expression on cells exposed to phthalimide for 72h, also using doses two times higher than those of lenalidomide, thus suggesting the involvement of CRBN in these mechanisms (Suppl. Fig. 5). Then, we tested whether the absence of CRBN could modify IMiDs effects on ligand expression. To this aim, we used a lentiviral vector expressing CRBN shRNA to generate SKO-007(J3) cells with reduced expression of this protein. Since stable CRBN depletion is cytotoxic for myeloma cells [31], these experiments were performed using transient infections. As shown in Fig. 3A–3C, lenalidomide failed to augment MICA and PVR/CD155 levels in cells with reduced levels of CRBN.

In order to identify possible molecular mediators involved in the upregulation of MICA and PVR/CD155 by IMiDs, we focused our attention on the role of the transcription factors Ikaros (IKZF1) and Aiolos (IKZF3), recently identified as IMiDs-bound CRBN downstream targets in MM cells [34-36].

We first confirmed the capability of lenalidomide and pomalidomide to promote IKZF1 and IKZF3 degradation in our experimental system (Suppl. Fig. 6A–6D); then, we examined whether the absence of these proteins could affect MICA and PVR/CD155 basal expression. To this purpose, we infected SKO-007(J3) cells with lentiviral vectors expressing IKZF1 or IKZF3 shRNAs along with green fluorescent protein (GFP) (Suppl. Fig. 7A and 7B). As shown in Fig. 3D–3G, by comparing ligand expression of shRNA-transduced (GFP^+^) and shRNA-non-transduced (GFP^−^) cells (Suppl. Fig. 7C), we could appreciate how knockdown of IKZF1 or IKZF3 proteins was sufficient to augment MICA and PVR/CD155 surface levels, suggesting a repressive role of these transcription factors on basal expression of these ligands in MM cells, whereas the expression of MICB was not affected.

To better evaluate and confirm these results, we infected SKO-007(J3) cells with a retrovirus expressing IK6-DN along with GFP, an isoform of Ikaros lacking the DNA binding domain and able to negatively interfere with the activity of wild-type Ikaros family members [43, 44]. Flow cytometric analysis on these cells revealed a significant increase of basal membrane expression of MICA and PVR/CD155 as compared to cells infected with an empty control vector (Suppl. Fig. 8A–8C). To determine the effects of IK6-DN on the expression of MICA and PVR/CD155 transcripts, GFP^+^ infected cells were isolated by cell sorting, for RNA extraction and Real-Time qRT-PCR analysis. Consistent with FACS analysis, overexpression of IK6-DN was able to increase MICA and PVR/CD155 basal mRNA levels (Suppl. Fig. 8D); moreover, as observed in IKZF1 or IKZF3 silenced cells, both MICB surface levels and transcripts were unaltered by ectopic expression of IK6-DN. Accordingly, we also observed significantly lower MICA and PVR/CD155 surface levels in wt-IKZF1-transduced 293T cells, that express IKZF1 at very low levels (Suppl. Fig. 8E, 8F).

**Figure 4:**
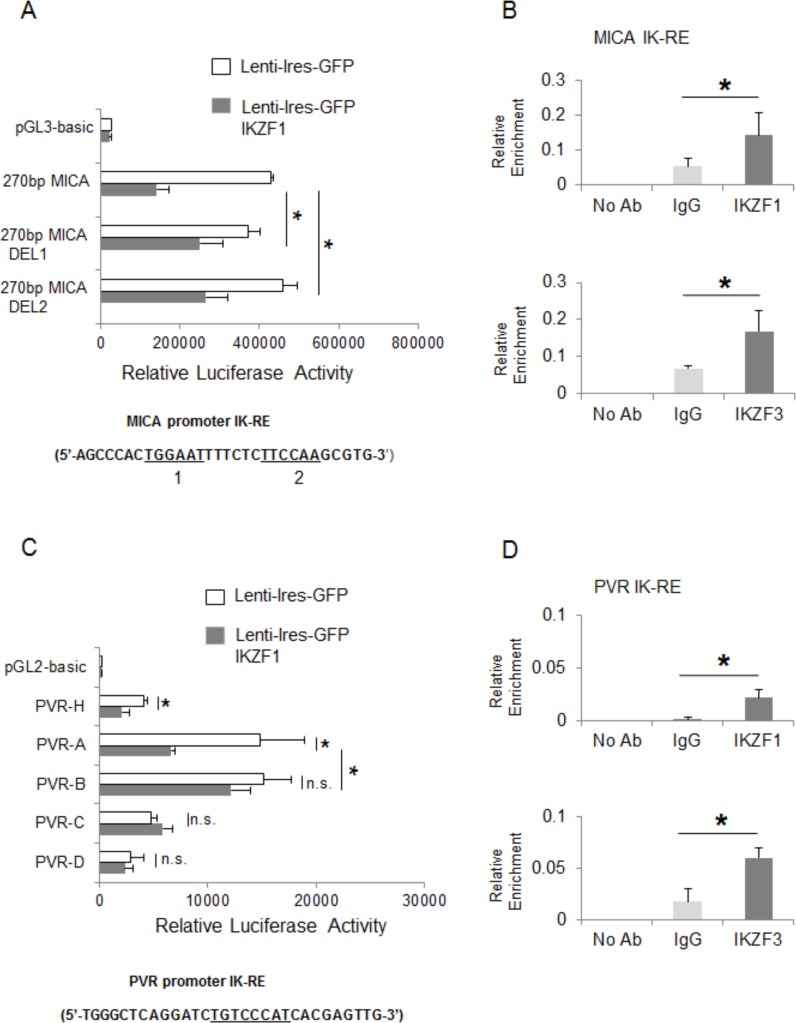
IKZF1/3 transcription factors repress mica and pvr promoter activity **A.**, **C.** 293T cells were transiently co-transfected with the indicated −270bp-*mica* reporter vectors or different *pvr* promoter deletions and an IKZF1 expression vector as described in Materials and Methods. After 48h, cells were harvested and protein extracts were prepared for the luciferase assay. Data were normalized to protein concentration and *β*-galactosidase activity and represent the mean of three independent experiments (*P < 0.05). **B.**, **D.** *In vivo* binding of IKZF1 and IKZF3 to the MICA and PVR promoters was examined in ChIP analysis. Samples were immunoprecipitated from sonicated lysates of SKO-007(J3) cells using an antibody against human IKZF1 or IKZF3 proteins or isotype control. After reversing the cross-linking, DNA was precipitated and Real-Time PCR was performed using primers to amplify the *mica* or *pvr* promoter region encompassing the Ikaros responsive element (IK-RE) (indicated in the figures). Results are expressed as Relative Enrichment as compared to the input. Data represent the mean of n=3 experiments (*P < 0.05). IK-RE: Ikaros Responsive Element.

To examine possible inhibitory effects of these transcription factors on *mica* and *pvr* promoter activity, we performed transient transfection assays in 293T cells. Regarding *mica*, a search for sequence homology revealed two proximal putative Ikaros response elements in a region spanning the first −270 bp of its promoter (Table 3). We observed a significant repressive effect mediated by IKZF1 overexpression on the luciferase activity driven by a −270 bp 5′-flank of *mica* promoter. However, the inhibitory effect of IKZF1 was significantly reduced on two mutated versions of the −270 bp *mica* promoter (indicated as 270bp MICA-DEL1 and 270bp MICA-DEL2), in which either one putative Ikaros site was removed by site-directed mutagenesis (Fig. 4A), thus indicating that binding of this transcription factor to both sites is required for repression.

**Table 3:**
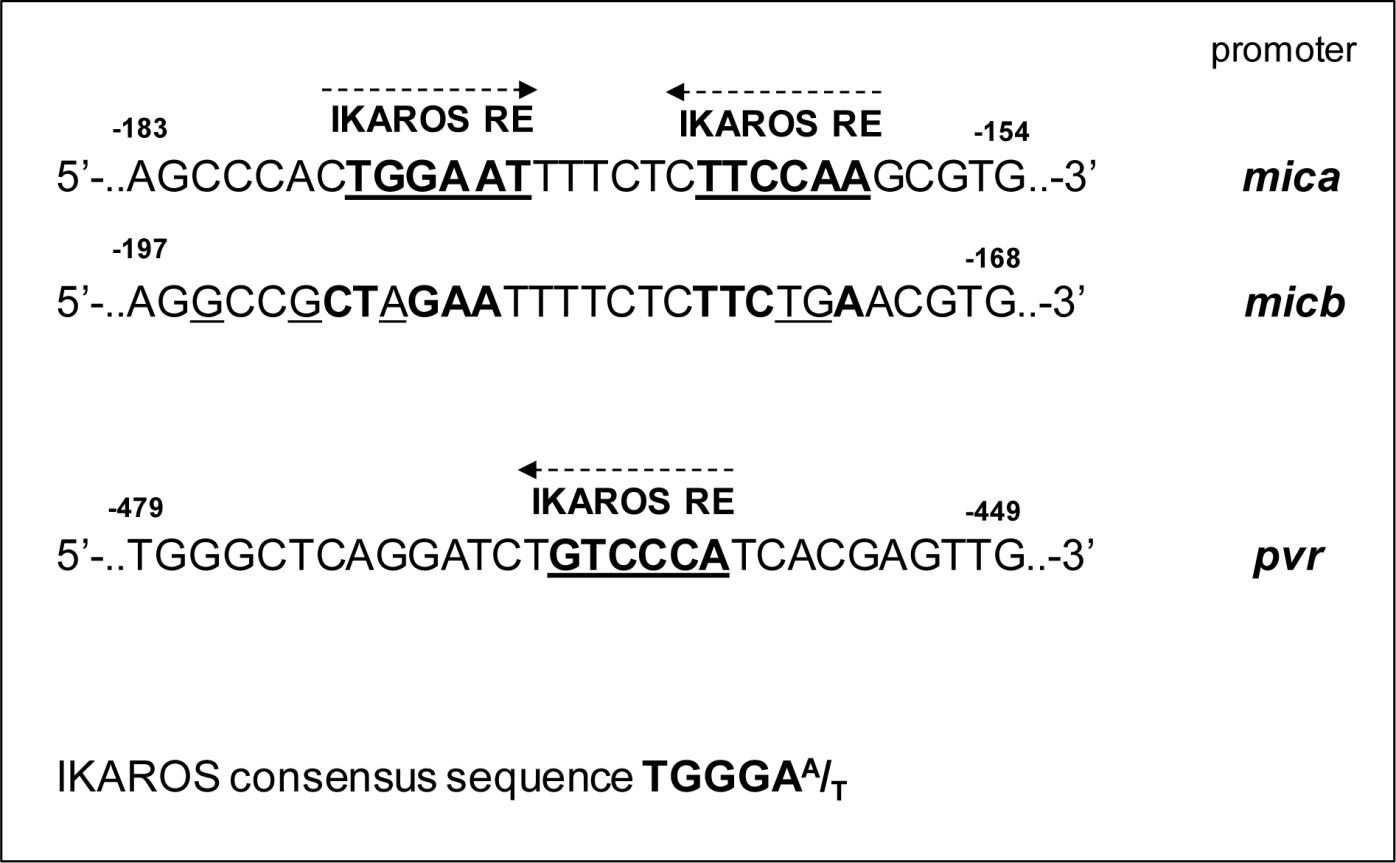
Schematic representation of putative IKAROS response elements of mica and pvr promoter

Regarding *pvr,* we used progressive deletions of this promoter and delineated a fragment, spanning from −470 bp to −343 bp (deletion PVR-A/PVR-B), responsive to the inhibitory activity of this transcription factor and containing a putative Ikaros consensus site (Fig. 4C).

Accordingly, we demonstrated the capability of IKZF1 and IKZF3 proteins to directly bind to *mica* and *pvr* promoters, in the regions encompassing these Ikaros consensus sites by ChIP assays (Fig. 4B and 4D).

**Figure 5:**
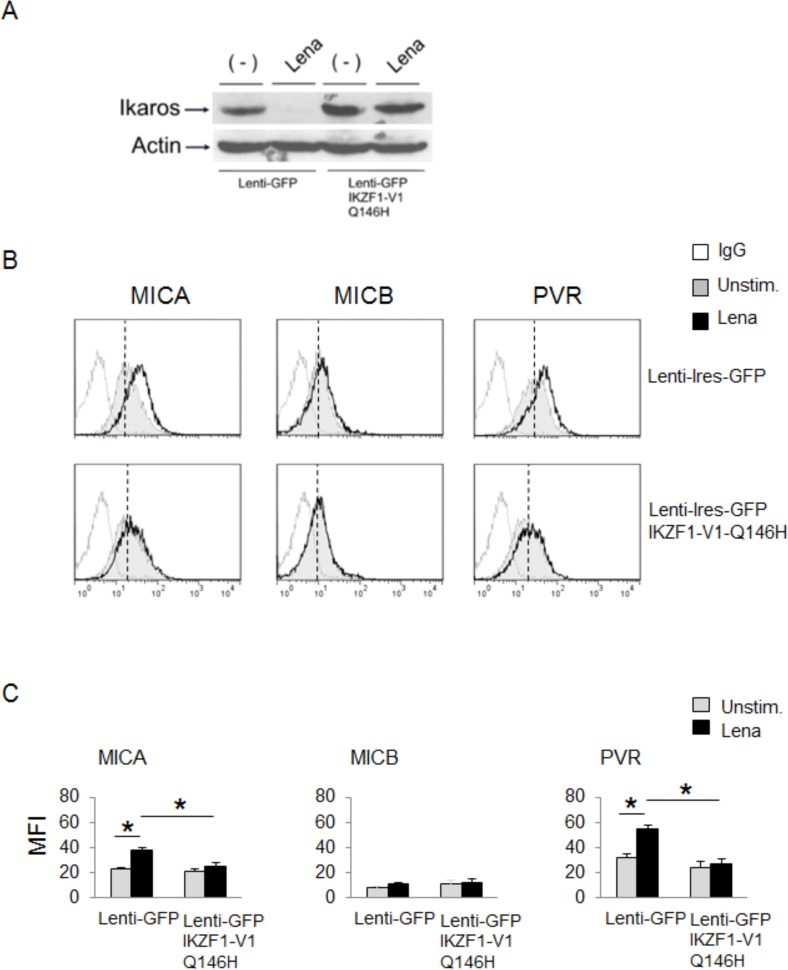
A mutant form of Ikaros incapable of binding CRBN abrogates MICA and PVR/CD155 upregulation by IMiDs **A.** Western Blot analysis of total cellular proteins from SKO-007(J3) cells infected with a control empty Lenti-Ires-GFP vector or Lenti-Ires-GFP-IKZF1-V1-Q146H, left unstimulated or stimulated with lenalidomide (Lena) for 72h. The proteins transferred to nitrocellulose membranes were stained with Ponceau to verify that similar amounts of proteins had been loaded in each lane and immunoblotted for IKZF1 and Actin. **B.** MICA, MICB and PVR/CD155 surface expression were analyzed by flow cytometry on SKO-007(J3) cells transiently infected with lentiviral vectors encoding GFP or GFP and IKZF1-V1-Q146H, untreated or treated with lenalidomide (Lena) as described above. Data are representative of one out of five independent experiments. The grey colored histograms represent basal expression of the indicated ligand, while thick black histograms represent the expression after treatment with lenalidomide (Lena). **C.** The MFI of MICA, MICB and PVR/CD155 were calculated based on at least five independent experiments and evaluated by paired Student *t* test (*P < 0.05). For each treatment, histograms represent the MFI of specific mAb - MFI of isotype control.

Finally, we did not observe any increase of ligand expression in lenalidomide-treated SKO-007(J3) cells overexpressing IKZF1-Q146H, a mutant form of Ikaros incapable of binding CRBN and resistant to IMiDs-mediated degradation [34] (Fig. 5A–5C), thus confirming the repressive effect of IKZF1/3 on MICA and PVR/CD155 expression, and indicating that IMiDs can blunt this effect.

### The transcription factor IRF4 inhibits MICA expression in MM cells

An additional target of IMiDs in MM cells is IRF4, a transcription factor positively regulated by IKZF1/3 and inhibited by IMiDs/CRBN-mediated degradation of these proteins [34-36].

We investigated its possible involvement in ligand upregulation, as no evidence is available about a role of this transcription factor in the regulation of MICA and PVR/CD155 gene expression.

**Figure 6:**
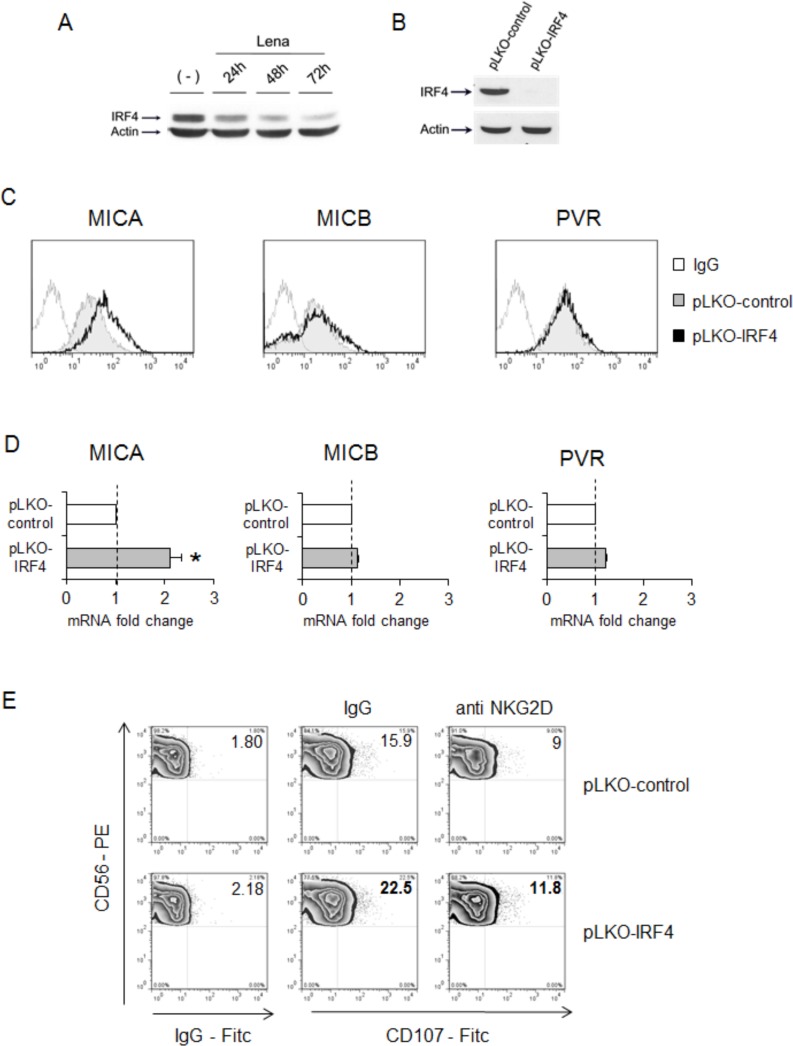
IRF4 represses MICA expression **A.** Lysates of SKO-007(J3) cells untreated or treated with lenalidomide (Lena) for 24h, 48h and 72h were subjected to Western Blotting using anti-IRF4 and Actin antibodies. The proteins transferred to nitrocellulose membranes were stained with Ponceau to verify that similar amounts of proteins had been loaded in each lane. Data are representative of one out of three independent experiments. **B.** Immunoblotting analysis for IRF4 and Actin of total cellular proteins from SKO-007(J3) cells infected with two different lentiviruses, pLKO expressing IRF4-shRNA or pLKO non-targeting shRNA (control) for 72h. The proteins transferred to nitrocellulose membranes were stained with Ponceau to verify that similar amounts of proteins had been loaded in each lane. Data are representative of one out of three independent experiments. **C.** MICA, MICB and PVR/CD155 surface expression were analyzed by flow cytometry on pLKO-control (non-targeting) or pLKO-IRF4-lentivirus infected SKO-007(J3) cells (72h). Data are representative of one out of four independent experiments. The grey colored histograms represent the expression of the indicated ligand in cells transduced with the pLKO-control, while thick black histograms represent the expression in cells infected with pLKO-IRF4. **D.** Total RNA were also isolated from infected cells for Real-Time qRT-PCR analysis. Data, expressed as fold change units, were normalized with GAPDH and referred to the cells infected with non-target shRNA, considered as calibrator and represent the mean of 3 experiments (*P < 0.05). **E.** NK cells prepared from PBMCs of healthy donors were incubated with SKO-007(J3) cells after 72h-infection with pLKO-control (non-targeting) or pLKO-IRF4 shRNA and used as target cells in a degranulation assay. The assay was performed as described above. Results are representative of one out of three independent experiments.

Consistent with other studies [34-36, 45], western blot analysis confirmed a significant IRF4 downregulation in SKO-007(J3) cells exposed to lenalidomide or pomalidomide (24 to 72h) (Fig. 6A and Suppl. Fig. 9). Therefore, we assessed the possible impact of direct IRF4 depletion by shRNA interference (Fig. 6B) on MICA and PVR/CD155 ligand expression. Since stable IRF4 depletion is cytotoxic for myeloma cells [40], these experiments were performed using transient infections.

Compared with non-targeting shRNA-infected cells, IRF4 shRNA-transduced cells expressed higher MICA surface levels, whereas MICB and PVR/CD155 membrane expression were unaffected (Fig. 6C). Accordingly, Real-Time qRT-PCR analysis showed that IRF4 silencing enhances only MICA mRNA expression (Fig. 6D). Moreover, we observed that the increased MICA membrane expression in IRF4 shRNA-transduced cells could enhance their capability to stimulate NK cell degranulation in an NKG2D-dependent manner (Fig. 6E).

These data indicate that in MM cells, IRF4 can repress MICA expression both at protein and mRNA levels.

**Figure 7:**
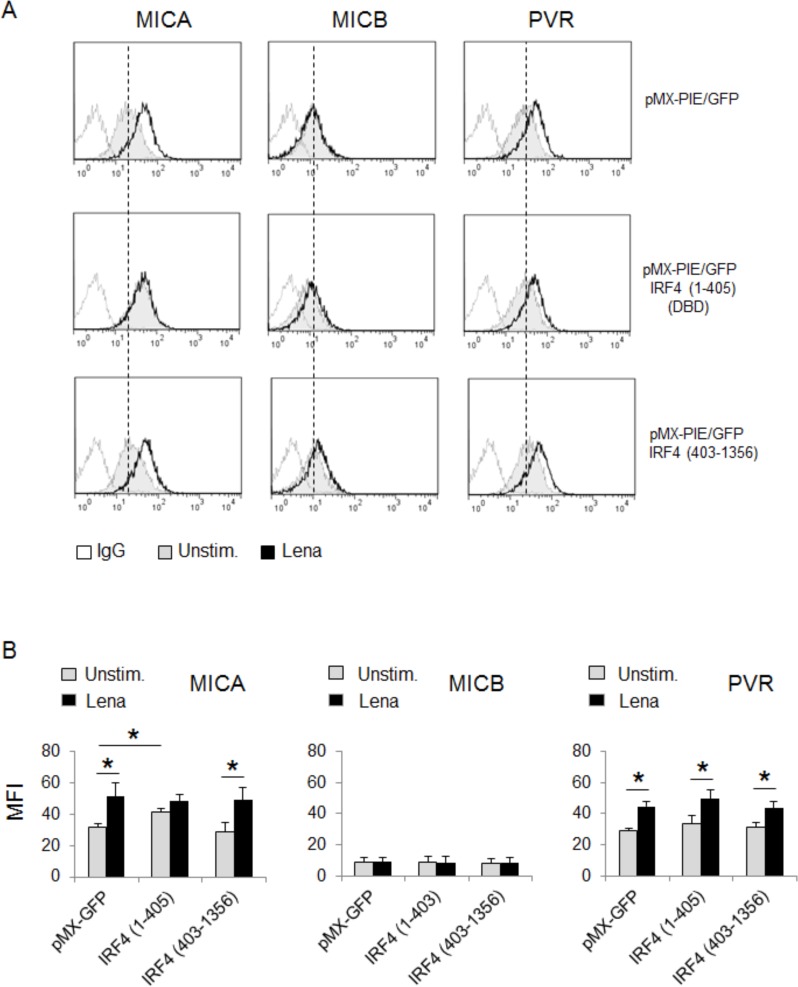
MICA inhibition by IRF4 depends on its DNA binding activity **A.** MICA and PVR/CD155 surface expression were analyzed by flow cytometry on SKO-007(J3) cells transduced with a retrovirus expressing IRF4 (1-405) or IRF4 (403-1356) and GFP, after 72h treatment with lenalidomide (Lena). Data are representative of one out of three independent experiments. The grey colored histograms represent basal expression of the indicated ligand, while thick black histograms represent the expression after treatment with the drug in GFP positive cells. **B.** The MFI of MICA, MICB and PVR/CD155 were calculated based on at least four independent experiments and evaluated by paired Student *t* test (*P < 0.05). For each treatment, histograms represent the MFI of specific mAb - MFI of isotype control.

To gain insight into the molecular mechanism(s) that mediate the repressive activity of IRF4 on MICA expression, we performed a structure/function analysis of IRF4 by infecting SKO-007(J3) cells with retrovirus expressing mutant forms of IRF4 and GFP. As shown in Fig. 7A and 7B, ectopic expression of a truncated form of IRF4 consisting of its N-terminal DNA binding domain (IRF4 1-405) (GFP^+^ cells) was sufficient to specifically increase MICA expression, presumably by competing with and inhibiting the endogenous wild-type IRF4 function. On the contrary, overexpression of the C-terminal association domain of IRF4 (IRF4 403-1356) did not affect either basal or lenalidomide-induced levels of this ligand, suggesting that DNA binding activity of IRF4 is necessary but not sufficient for MICA repression.

These observations indicate that IMiDs may involve both IKZF1/3 proteins depletion and IRF4 downregulation into increased MICA expression.

**Figure 8:**
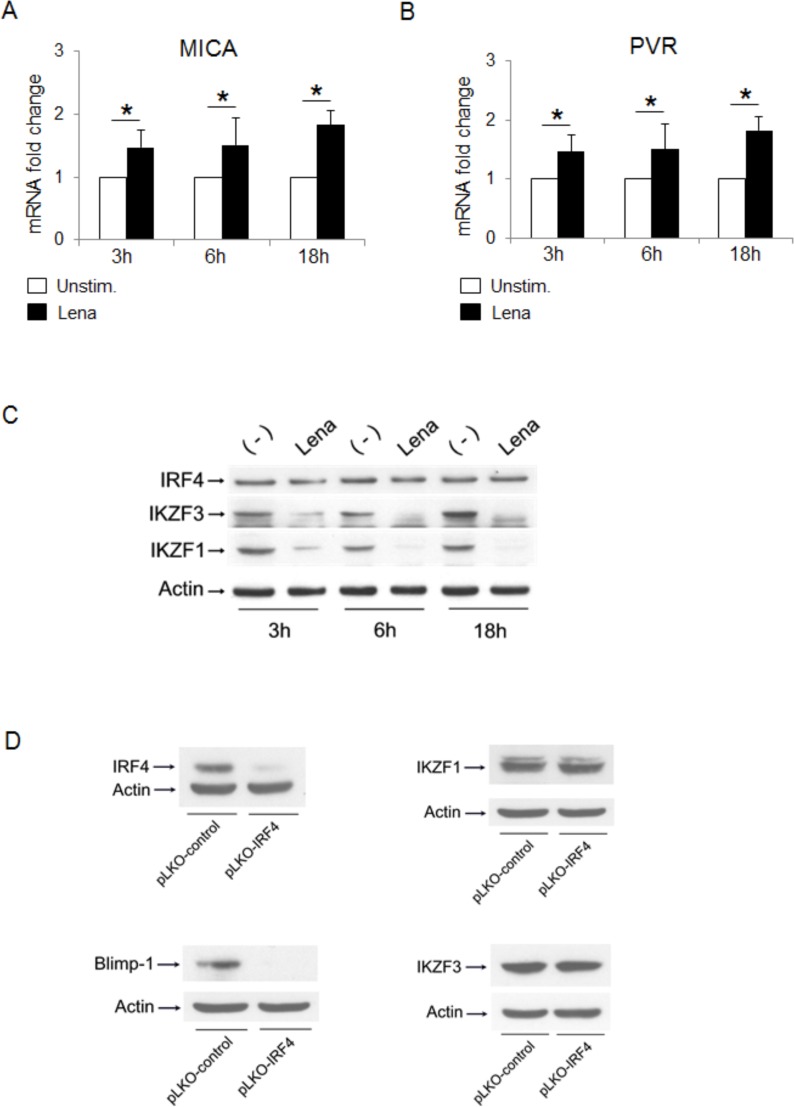
Loss of the transcription factors IKZF1/3 and IRF4 contributes to IMiDs-mediated MICA upregulation with different kinetics **A.**, **B.** Real Time PCR analysis of total mRNA obtained from SKO-007(J3) cells, unstimulated or treated with lenalidomide (Lena) for the indicated times. Data, expressed as fold change units, were normalized with GAPDH and referred to the untreated cells considered as calibrator and represent the mean of 3 experiments (*P < 0.05). **C.** SKO-007(J3) cells were treated with lenalidomide (Lena) for the indicated times and cell lysates were immunoblotted for IRF4, IKZF1, IKZF3 and Actin. The proteins transferred to nitrocellulose membranes were stained with Ponceau to verify that similar amounts of proteins had been loaded in each lane. Data are representative of one out of three independent experiments. **D.** Immunoblotting analysis for IRF4, Blimp-1, IKZF1, IKZF3 and Actin of total cellular proteins from SKO-007(J3) cells infected with a lentivirus expressing IRF4 shRNA or pLKO-control (non-targeting) shRNA. The proteins transferred to nitrocellulose membranes were stained with Ponceau to verify that similar amounts of proteins had been loaded in each lane. Data are representative of one out of three independent experiments.

Interestingly, by analyzing the induction of ligands at early times, treatment with lenalidomide was able to increase MICA and PVR/CD155 mRNA levels as early as 3 to 18 h (Fig. 8A and 8B), inducing IKZF1 and IKZF3 degradation with no significant effect on IRF4 levels (Fig. 8C), thus suggesting that IKZF1/3 and IRF4 could contribute to MICA upregulation through a time-sequential modulation of their expression.

Finally, knockdown of IRF4 did not modify either IKZF1 or IKZF3 expression, while it could abolish the expression of Blimp-1 (a known target used here as a control) [46] (Fig. 8D), thus excluding the possibility that the IRF4-mediated negative regulation of MICA might indirectly depend on IKZF1/3 transcription factors [47].

Altogether, these observations identify the transcription factor IRF4 as a novel negative regulator of MICA gene expression in MM cells and provide evidence that its downregulation contributes to MICA upregulation by IMiDs.

## DISCUSSION

IMiDs are highly effective drugs for the treatment of MM and different biological activities are responsible for their anti-myeloma properties [21, 22, 48, 49]. In particular, a number of evidences in myeloma patients describe the anti-tumor potential of NK cells in response to IMiDs [19, 20, 23, 24], as well as the sensitization of myeloma cells to NK cell-mediated lysis [27, 28].

In this study, we investigated the effects of IMiDs on NK cell-activating ligand expression in MM cells. We found a significant upregulation of MICA and PVR/CD155 mRNA and cell surface expression by lenalidomide and pomalidomide; accordingly, NK-cell degranulation was enhanced upon interaction with IMiDs-treated tumor cells.

IMiDs bind to CRBN, a component of the ubiquitin ligase complex CLR4; the drug-CRBN interaction alters its specificity to induce ubiquitylation and degradation of several proteins, involved in the regulation of different molecular pathways [34-36]. In this regard, the amount of three different transcription factors, IKZF1, IKZF3 and IRF4 was widely described to decrease in MM cells exposed to IMiDs.

Our findings demonstrate that IMiDs-CRBN-IKZF1/3-IRF4 axis is critically involved in NK cell-activating ligand regulation by IMiDs in MM cells.

We show that CRBN knockdown can abrogate the capacity of lenalidomide to increase MICA and PVR/CD155 expression, further supporting the role of this protein in anti-MM actions of this drug.

Moreover, we provide evidence that the transcription factors IKZF1 and IKZF3 repress basal MICA and PVR/CD155 expression in MM cells; our data indicate that silencing of IKZF1 or IKZF3 by shRNA interference (Fig. 3D–3G) or inhibition of their activity by IK6-DN overexpression (Suppl. Fig. 8A–8D) is sufficient to increase MICA and PVR/CD155 expression, both at protein and mRNA levels.

IKZF1 and IKZF3 transcription factors are able to bind the same regulatory elements of their target genes, either as homo- or heterodimers [35, 38]. We observed that knockdown of IKZF1 or IKZF3 results in a similar increase of NK cell-activating ligand expression, thus indicating that both transcription factors can suppress their expression.

We also identified a region of the *pvr* promoter containing an Ikaros response element and interacting with IKZF1 and IKZF3, supporting a direct involvement of these proteins in the transcriptional regulation of this ligand (Fig. 4C, 4D). Moreover, a site-directed mutagenesis revealed the involvement of two different proximal regions in the repression of *mica* promoter by IKZF1/3, and ChIP analysis showed that both IKZF1 and IKZF3 directly interact with this promoter *in vivo* (Fig. 4A, 4B). Interestingly, Ikaros consensus sites overlap a region of *mica* promoter, previously identified as an heat shock response element (HSE), critical for the induction of this promoter, as well as *micb* promoter, by different stress conditions via HSF1 activation [17, 50]. Of note, *mica* and *micb* promoters are highly homologous, yet our data indicate a different regulation of these ligands in MM cells. Indeed, we did not observe a significant stimulatory effect of IMiDs or IKZF1/3 silencing on MICB expression, both at protein and mRNA levels, either in MM cell lines or primary malignant plasma cells; a possible explanation could be that Ikaros response elements are not conserved in *micb* promoter (Table 3).

IMiDs can epigenetically regulate different genes in MM cells [36, 51, 52], and histone deacetylase (HDAC) or DNA methylation inhibitors can induce NKG2D ligand expression in different cancer cells [53-56]; moreover, a possible role of epigenetic mechanisms in the regulation of PVR/CD155 expression has been also described [57]. In this regard, IKZF1/3 transcription factors can promote gene repression or activation through their ability to recruit, via their C-terminus, a large number of nuclear co-factors, including components of both HDAC and ATP-dependent chromatin remodeling complexes [58-60]. Thus, epigenetic modifications likely can contribute to IKZF1/3-dependent repression of *mica* and *pvr* promoter activity. Further experiments will be needed to address this point.

In addition, our data indicate that *mica* promoter activity can be also indirectly repressed by IKZF1/3 via IRF4 [34-36], another transcription factor emerged in our study as a novel negative repressor of MICA expression. Indeed, IRF4 silencing was sufficient, per sé, to significantly and specifically increase MICA expression, both at protein and mRNA levels (Fig. 6).

IRF4 can positively and negatively regulate different genes, in part, by binding to distinct DNA-binding motifs and through interaction with various additional transcription factors [61]; its C-terminal transactivation domain is critical for gene activation, while the mechanisms responsible for gene repression are not well defined.

Here, we demonstrated that the DNA binding activity of IRF4 is necessary for its inhibitory role on MICA expression (Fig. 7); in this regard, by computer analysis of the hMICA 5′-flank, we could identify several putative IRF4 binding sites. Our data tend to rule out a possible regulation of IKZF1 and IKZF3 proteins by IRF4 (Fig. 8), and suggest that the repressive effect of this transcription factor on MICA expression occurs via IKZF1 and IKZF3 independent mechanisms. Further studies will be needed to better clarify a direct or indirect repressive role of IRF4 on *mica* promoter.

In conclusion, we propose a model in which IKZF1/3 can repress the constitutive expression of MICA and PVR/CD155 expressed on MM cells, while IRF4 is able to inhibit only MICA.

IMiDs promote the CRBN-dependent degradation of IKZF1/3 proteins, leading to de-repression of *pvr* promoter and to an initial de-repression of *mica* promoter, further enhanced when also IRF4 is downregulated. In this context, further experiments will be necessary to investigate the possibility that these regulatory mechanisms could be involved also in other cell lines (e.g. lymphoma, AML, MDS).

These findings provide new insights on the immuno-mediated antitumor activities of IMiDs and further elucidate the molecular mechanisms that regulate NK cell-activating ligand expression.

## MATERIALS AND METHODS

### Cell lines and clinical samples

Human myeloma cell lines SKO-007(J3), ARP-1 and OPM-2 were kindly provided by Prof. P. Trivedi (University of Rome, Sapienza, Italy). The human MM cell lines JJN-3 and KMS-27 were kindly provided by Prof. N. Giuliani (University of Parma, Italy). These cell lines were maintained at 37°C and 5% CO_2_ in RPMI 1640 supplemented with 10% FCS, 2 mM L-glutamine, 100 U/ml penicillin and 100 U/ml streptomycin (complete medium). The human 293T embryonic kidney cells were purchased from ATCC and were maintained in Dulbecco's modified Eagle's supplemented with 10% FCS. All cell lines were mycoplasma-free (EZ-PCR Mycoplasma Test Kit, Biological Industries).

Bone marrow samples from patients with MM were managed at the Division of Hematology, Department of Cellular Biotechnologies and Hematology, University of Rome, Sapienza, Italy. Informed consent in accordance with the Declaration of Helsinki was obtained from all patients, and approval was obtained from the Ethics Committee of the Sapienza University of Rome. The bone marrow aspirates were processed as already described in [62]. In some experiments, myeloma cells were selected using anti-CD138 magnetic beads (Miltenyi Biotec). More than 95% of the purified cells expressed CD138 and CD38.

### Reagents and antibodies

Lenalidomide was purchased from (BioVision Inc.), Pomalidomide and Phthalimide were purchased from (Sigma-Aldrich). These drugs were dissolved in dimethylsulphoxide (DMSO) and stored at −20°C until use. The final concentration of DMSO in all experiments was < 0.1%.

The following monoclonal antibodies (mAbs) were used for immunostaining or as blocking Abs: anti-MICA (MAB159227), anti-MICB (MAB236511), anti-ULBP-1 (MAB170818), anti-ULBP-2/5/6 (MAB165903), anti-ULBP-3 (MAB166510) and anti-NKG2D (MAB149810) from (R&D System), anti-PVR/CD155 (SKII.4) kindly provided by Prof. M. Colonna (Washington University, St Louis, MO), anti-CD56 (C218) mAb was provided by Dr. A. Moretta (University of Genoa, Genoa, Italy), anti-DNAM-1 (DX11) from (Serotec), APC Goat anti-mouse IgG (Poly4053), anti-CD3/APC (HIT3a), anti-CD56/PE (HCD56), mouse IgG1/FITC, /PE or /APC isotype control (MOPC-21) were purchased from (BioLegend). Anti-CD107a/FITC (H4A3), anti-CD138-FITC (M15) and anti-CD38-APC (HIT2) were purchased from (BD Biosciences).

### Flow cytometry and degranulation assay

SKO-007(J3), ARP-1, OPM-2, JJN-3 and KMS-27 cells were cultured in 6-well tissue culture plates for 72h at a concentration of 2 × 10^5^ cells/ml in the presence of the indicated drug. The expression of the NKG2D and DNAM-1 ligands on MM cells was analyzed by immunofluorescence staining using anti-MICA, anti-MICB or anti-PVR/CD155 unconjugated mAb, followed by secondary GAM-APC. In all experiments, cells were stained with Propidium Iodide (PI) (1 μg/ml) in order to assess cell viability (always higher than 90% after the different treatments). Nonspecific fluorescence was assessed by using an isotype-matched irrelevant mAb (R&D System) followed by the same secondary antibody. Fluorescence was analyzed using a FACSCalibur flow cytometer (BD Biosciences) and data were analyzed using FlowJo Flow Cytometric Data Analysis Software (Tree Star, Inc.).

NK cell-mediated cytotoxicity was evaluated using the lysosomal marker CD107a as previously described [17]. As source of effector cells, we used primary NK cells obtained from PBMCs isolated from healthy donors by Lymphoprep (Nycomed) gradient centrifugation and then co-cultured for 10 days with irradiated (30 Gy) Epstein-Barr virus (EBV)-transformed B-cell line RPMI 8866 at 37°C in a humidified 5% CO_2_ atmosphere, as previously described [17]. On day 10, the cell population was routinely more than 90% CD56^+^CD16^+^CD3^−^, as assessed by immunofluorescence and flow cytometry analysis. When patient-derived plasma cells were used as targets, autologous CD138^−^ bone marrow cells were cultured for 2 days in complete medium, supplemented with 200 U/mL IL-2, and used as source of effector cells.

Drug-treated MM cell lines or patient-derived plasma cells were washed twice in complete medium and incubated with NK cells at effector:target (E:T) ratio of 2.5:1, in a U-bottom 96-well tissue culture plate in complete medium at 37°C and 5% CO2 for 2 h. Thereafter, cells were washed with PBS and incubated with anti-CD107a/FITC (or cIgG/FITC) for 45 min at 4°C. Cells were then stained with anti-CD3/APC, anti-CD56/PE and anti-CD16/PerCP-Cy5.5 to gate the CD3^−^CD56^+^ CD16^+^ NK cell population. In some experiments, cells were pre-treated for 20 min at room temperature with anti-NKG2D or anti-DNAM-1 neutralizing mAbs. Fluorescence was analyzed using a FACSCalibur flow cytometer (BD Biosciences) and data were analyzed using FlowJo Flow Cytometric Data Analysis Software (Tree Star).

### Plasmids

For knocking down CRBN and IRF4 we used a pLKO.1-sh-Cereblon (TRCN0000141562) or a pLKO.1-sh-IRF4 (TRCN0000014764) lentiviral vector and the control vector pLKO non-targeting shRNA (MISSION™ Sigma-Aldrich). For knocking down IKZF1 and IKZF3 we used a pLKO.1-sh-IKZF1 (TRCN0000236420) or a IKZF3 (TRCN0000414188) GFP-lentiviral vector (MISSION™ Sigma-Aldrich). The retroviral vectors pMYs-IRES-EGFP and pMYs-IK6-IRES-EGFP, encoding a dominant negative isoform of Ikaros transcription factors, were kindly provided by Dr. Nosaka T. (Graduate School of Medicine, Mie University, Japan) [44]. The lentiviral vectors CAG-IRES-GFP encoding IKZF1 were kindly provided by Dr. Kaelin W.G. (Dana-Farber Cancer Institute, Boston, MA, USA) [34]. The lentiviral vectors MICA (HPRM 15126-LvPG04) and PVR/CD155 (HPRM 13303-LvPG04) promoter reporter and NEG-LVPG04 negative control were purchased from (GeneCopoeia Inc., Rockville, MD). The retroviral vectors pMX-IRF4 (1-405) and pMX-IRF4 (403-1356), encoding truncated forms of human IRF4 consisting of its N-terminal DNA binding domain and its C-terminal association domain respectively, were generated by inserting the mutant IRF4 cDNAs obtained from the expression vector pcDNA3-IRF4 (1-405) and pcDNA3-IRF4 (403-1356) (kindly provided by Dr. Hayashi H., Graduate school of medical Sciences, Nagasaki University, Japan) [63], in the retrovirus pMX/PIE-GFP. MICA-270 bp promoter in pGL3-basic luciferase vector (Promega Corp., Madison, WI) was generated as previously described [62]. The mutant MICA promoter constructs (270bp-MICA-DEL1 and 270bp-MICA- vector DEL2) were generated using Quick Change Site-Directed Mutagenesis Kit (Statagene) following the manufacturer's instructions. Four primers were designed to generate two different deletions of the Ikaros binding site (responsive element) in the MICA promoter region of the pGL3-270 bp MICA. The sequences were the following:

270 bp MICA prom. DEL1 sense, 5′-CTCCCCAGGTCTCCTTTTCTCTTCCAAGCG-3′:

270 bp MICA prom. DEL1 antisense 5′-CGCTTGGAAGAGAAAAGGAGACCTG GGGAG-3′;

270 bp MICA prom. DEL2 sense 5′-CTCCAGCCCACTGGAAGCGTGGCCCCGCC-3′;

270 bp MICA prom. DEL2 antisense 5′-GGCGGGGCCACGCTTCCAGTGGGCTGGAG-3′.

All constructs were verified by DNA sequence analysis.

The different deletions of the human PVR/CD155 promoter (in pGL2-basic luciferase vector, Promega Corp.) were kindly provided by Dr. Bernhardt G. (Hannover Medical School, Hannover, Germany) [64].

### DNA transfections, virus production and *in vitro* transduction

293T cells were transfected with 0.5 μg of MICA-270 bp/pGL3 basic or PVR/pGL2 basic luciferase reporter plus 0.125 μg of expression vector encoding IKZF1 using Lipofectamine Plus (Life Technologies). A RSV-gal expression vector was co-transfected to normalize DNA uptake. After 48h, cells were harvested and protein extracts were prepared for the luciferase and beta-galactosidase assays as already described in [65]. For MICA and PVR/CD155 lentiviral promoter vectors, Gaussia luciferase and secreted Alkaline Phosphatase activities were determined using Secrete-Pair™ Dual Luminescence Assay Kit (GeneCopoeia Inc.) and read using the Glomax Multi Detection System (Promega Corp.) following the manufacturer's instructions.

For retrovirus production, Phoenix cells were transfected with 5 μg of viral DNA using Lipofectamine Plus (Life Technologies). The lentiviral vectors were cotransfected together the packaging vectors pVSVG and psPAX2 into 293T cells using Lipofectamine Plus. After transfection, the cells were placed in fresh medium. After a further 48-hour culture, virus-containing supernatants were harvested, filtered, and used immediately for infections. Infections were performed on 0.5 × 10^6^ SKO-007(J3) cells in 2 ml complete medium with Polybrene (8 μg/ml) (hexadimethrine bromide; Sigma-Aldrich) for 2 h. For GFP-expressing viruses, the infection efficiency was measured by FACS analysis of GFP expression at day 3 after infection. In some experiments, SKO-007(J3) cells infected by retrovirus pMYs-IK6-IRES-EGFP or pMYs-IRES-EGFP were sorted based on GFP expression using a FACSAria (BD Biosciences) equipped with a 488nm laser and FACSDiva software (BD Biosciences version 6.1.3). Briefly, cells first gated based on forward and side scatter area (FSC-A and SSC-A) plot were then detected in the green fluorescence channel for GFP expression.

### RNA isolation, quantitative Real-Time polymerase chain reaction (qRT-PCR), and chromatin immunoprecipitation (ChIP) assay

Total RNA was extracted using TRIZOL^TM^ (Life Technologies), according to manufacturer's instructions. The concentration and quality of the extracted total RNA was determined by measuring light absorbance at 260 nm (A_260_) and the ratio of A_260_/A_280_. Reverse transcription was carried out in a 25 μl reaction volume with 2 μg of total RNA according to the manufacturer's protocol for M-MLV reverse transcriptase (Promega). Real-Time qRT-PCR was performed using TaqMan assays and the ABI Prism 7900 Sequence Detection system (Applied Biosystems). cDNAs were amplified in triplicate with primers for MICA (Hs00792195_m1), MICB (Hs00792952_m1), PVR/CD155 (Hs00197846_m1), CRBN (Hs00372271_m1) and GAPDH (Hs03929097_g1) conjugated with fluorochrome FAM (Applied Biosystems). The level of expression was measured using Ct (threshold cycle). The Ct was obtained by subtracting the Ct value of the gene of interest from the housekeeping gene (GAPDH) Ct value. In the present study, we used Ct of the untreated sample as the calibrator. The fold change was calculated according to the formula 2^−ΔΔCt^, where ΔΔCt was the difference between Ct of the sample and the Ct of the calibrator (according to the formula, the value of the calibrator in each run is 1). The analysis was performed using the SDS version 2.4 software (Applied Biosystems).

ChIP assays were done using Magna Chip kit protocol (EMD Millipore, Bedford, MA); after sonication, chromatin samples were immunoprecipitated overnight with 10 μg of a polyclonal rabbit anti-Ikaros (H-100), polyclonal goat anti-Aiolos antibody (L-15) (Santa Cruz Biotechnology) or isotype control and 20 μl of fully suspended protein A/G magnetic beads. The immunocomplexes/beads were washed sequentially with low-salt buffer, high-salt buffer, LiCl wash buffer and TE buffer, as detailed in the Upstate Biotechnology protocol. Precipitated were extracted with an elution buffer (1% SDS/0.1 M NaHCO_3_) and digested with proteinase K for 2 h at 62°C. DNA was purified using spin columns and eluted in 50 μl of DEPC-water. Fractions of pre-cleared chromatin (1%) were processed similar to immunoprecipitated chromatin, for input DNA controls.

The purified DNA was quantified in triplicate using Real-Time PCR and the Power-SYBR green mix with ROX (Applied Biosystems). Primer sequences were as follows:

MICA promoter forward: 5′-AGGTCTCCAGCCCACTGGAATTTTCTC-3′;

MICA promoter reverse: 5′-CGCCACCCTCTCAGCGGCTCAAGC-3′;

PVR promoter forward: 5′-CAGGATCTGTCCCATCACGAGTTGG-3′;

PVR promoter reverse: 5′-GCTCCGTCGGTGCCCACTAGA-3′.

PCRs were validated by the presence of a single peak in the melt curve analysis, and amplification of a single specific product was further confirmed by electrophoresis on agarose gel. Results are expressed as relative enrichment as compared to the input. Negative control (no antibody) values were subtracted from the corresponding samples. Absolute quantifications were obtained by serial dilutions of the input DNA samples. The analysis was performed using the SDS version 2.4 software (Applied Biosystems).

### Western-blot analysis

For Western-Blot analysis, SKO-007(J3) cells were pelleted, washed once with cold phosphate-buffered saline, resuspended in lysis buffer [1% Nonidet P-40 (v/v), 10% glycerol, 0.1% SDS, 0.5% Sodium Deoxycholate, 1 mM phenyl-methyl-sulfonyl fluoride (PMSF), 10 mM NaF, 1 mM Na3VO4, complete protease inhibitor mixture (Roche) in PBS] and subsequently incubated 30 min on ice. The lysate was centrifuged at 14000g for 15 min at 4°C and the supernatant was collected as whole cell extract. Protein concentration was determined by the BCA method (Pierce). Thirty to 50 μg of cell extract was run on 12.5% denaturing SDS-polyacrylamide gels. Proteins were then electroblotted onto nitrocellulose membranes (Schleicher&Schuell), stained with Ponceau to verify that similar amounts of proteins had been loaded in each lane, and blocked in 3% milk in TBST buffer. Immunoreactive bands were visualized on the nitrocellulose membranes, using horseradish-peroxidase-coupled goat anti-rabbit or goat anti-mouse immunoglobulins and the ECL detection system (GE Healthcare Amersham), following the manufacturer's instructions. Antibodies against *β*-actin, IRF4 (H-140), Ikaros (H-100) and Aiolos (L-15) were purchased from Santa Cruz Biotechnology. Antibody against Blimp-1 was purchased from Cell Signaling.

### Statistics

Error bars represent SD. Data were evaluated by paired Student *t* test and a level of *P* < 0.05 was considered statistically significant.

## SUPPLEMENTAL MATERIAL FIGURES


