# Supplementary material for: The IMiDs targets IKZF-1/3 and IRF4 as novel negative regulators of NK cell-activating ligands expression in multiple myeloma
Source: Oncotarget. 2015 Jun 23;6(27):23609–30. doi: 10.18632/oncotarget.4603 (PMC4695140; doi:10.18632/oncotarget.4603)
Supplement: Supplementary file 1 [file oncotarget-06-23609-s001.pdf]

The IMiDs targets IKZF-1/3 and IRF4 as novel negative regulators of NK cell-activating ligands expression in multiple myeloma

Supplementary Material

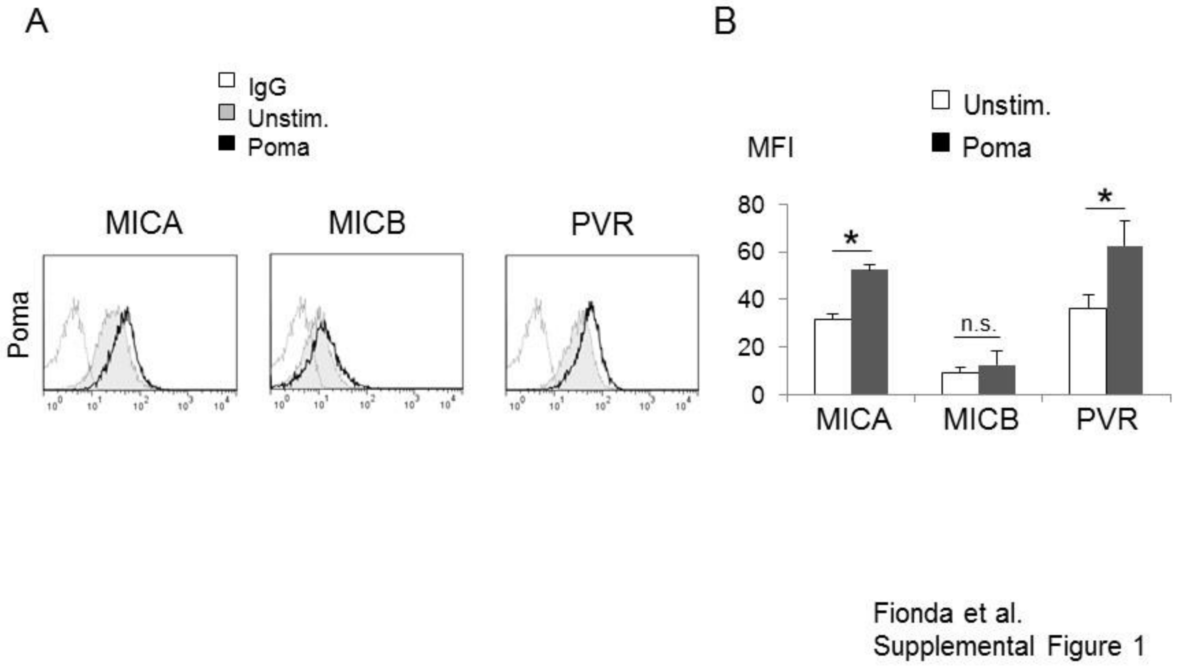

Supplemental Figure 1 - **IMiDs upregulate MICA and PVR/CD155 expression on human Multiple Myeloma cells.** A) MICA, MICB and PVR/CD155 surface expression were analyzed by flow cytometry on SKO-007(J3) cells treated with IMiDs, pomalidomide (Poma) (10 µM) for 72h. The grey colored histograms represent basal expression of the indicated ligand, while thick black histograms represent the expression after treatment with the drug. Data are representative of one out of four independent experiments. B) The MFI of MICA, MICB and PVR/CD155 were calculated based on at least four independent experiments and evaluated by paired Student *t* test (**P*< 0.05). Histograms represent the MFI of specific mAb - MFI of isotype control.

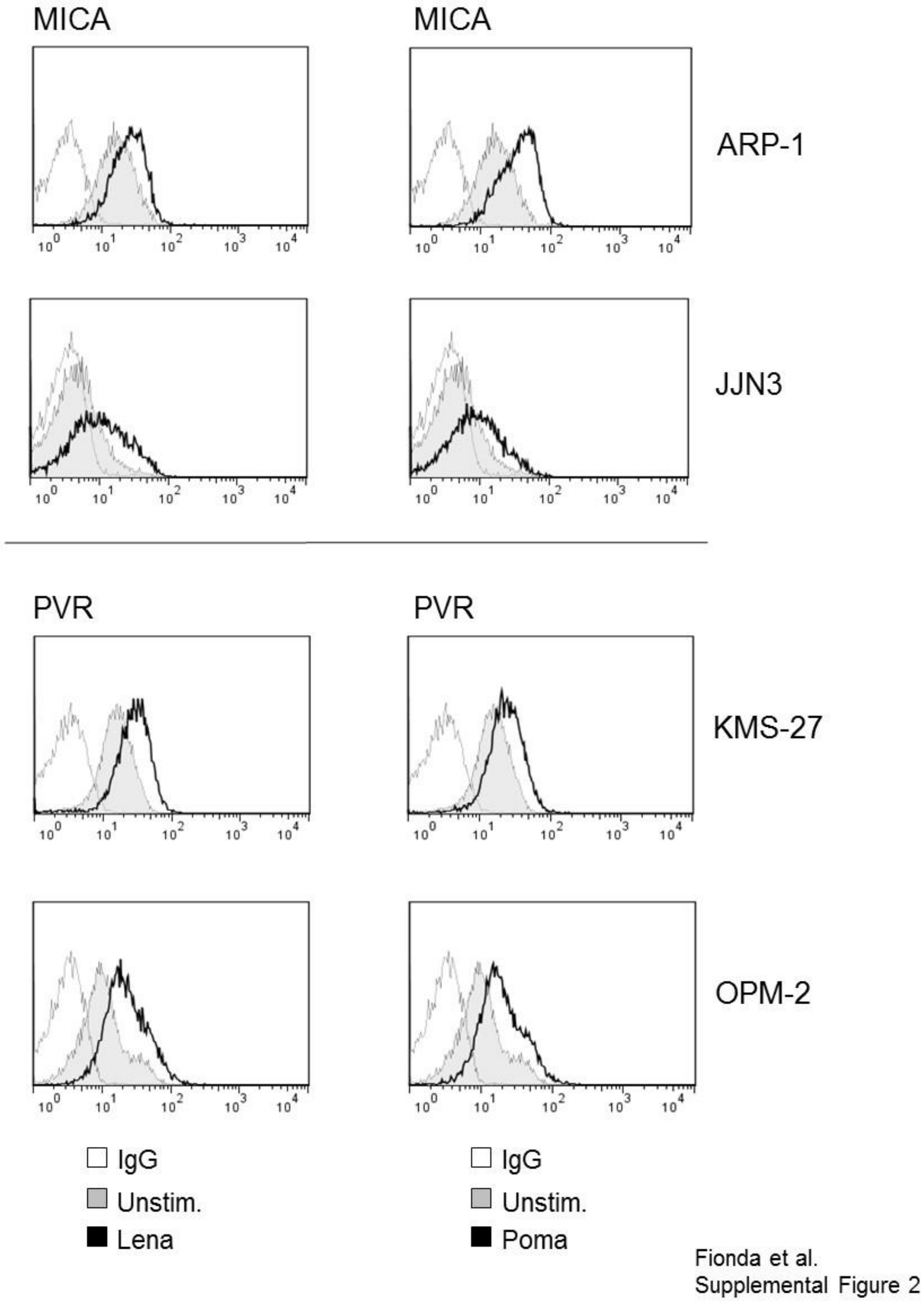

Supplemental Figure 2 - **Upregulation of MICA and PVR/CD155 expression on MM cell lines following treatment with IMiDs.** MICA and PVR/CD155 surface expression were analyzed by immunofluorescence and flow cytometry on ARP-1 and JJN3 or KMS27 and OPM-2 cells, respectively, after a 72h treatment with lenalidomide (Lena) or pomalidomide (Poma). The grey colored histograms represent basal expression of the indicated ligand, while thick black histograms represent the expression after treatment with the indicated drug. Data are representative of one out of three independent experiments.

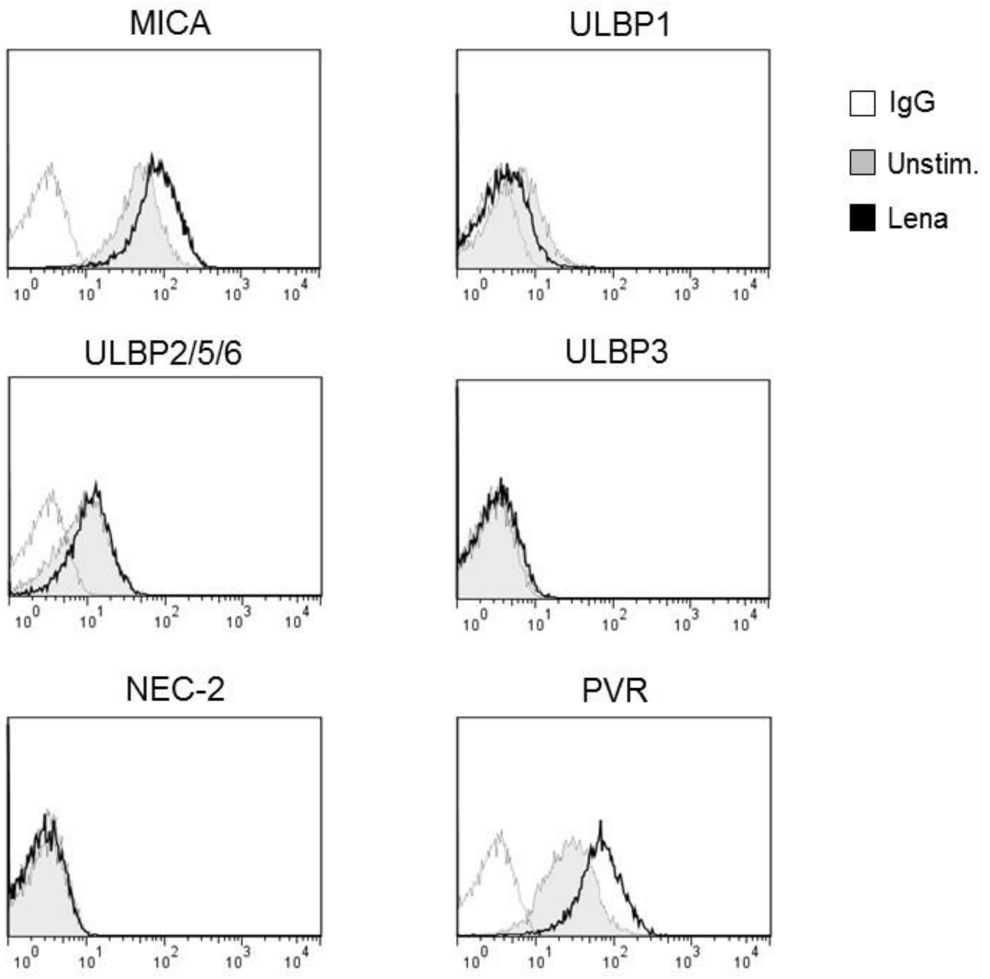

Fionda et al.
Supplemental Figure 3

Supplemental Figure 3 - **ULBPs and Nec-2 ligand expression on SKO-007(J3) cells following treatment with lenalidomide.** ULBPs and Nec-2 surface expression were analyzed by immunofluorescence and flow cytometry on SKO-007(J3) cells treated with lenalidomide (Lena) as described above for 72h. The grey colored histograms represent basal expression of the indicated ligand, while thick black histograms represent the expression after treatment with the drug. Data are representative of one out of three independent experiments.

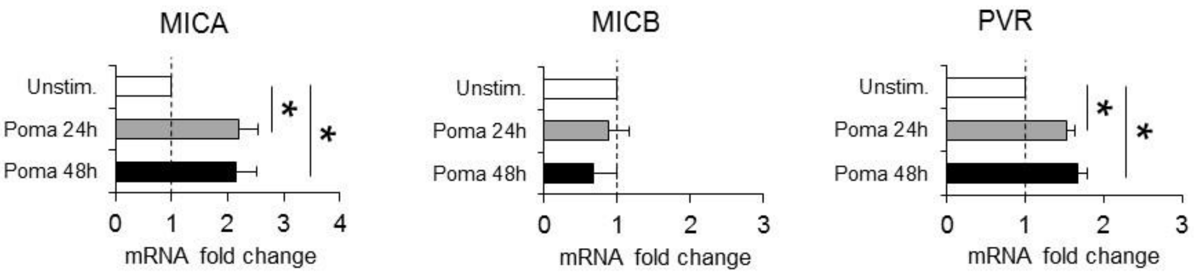

Fionda et al.
Supplemental Figure 4

Supplemental Figure 4 - **Pomalidomide increases MICA and PVR/CD155 mRNA expression in SKO-007(J3) cells.** Real Time PCR analysis of total mRNA obtained from SKO-007(J3) cells, untreated or treated with pomalidomide (Poma) as described above for 24h and 48h. Data, expressed as fold change units, were normalized with GAPDH and referred to the untreated cells, considered as calibrator and represent the mean of 3 experiments (*$P < 0.05$).

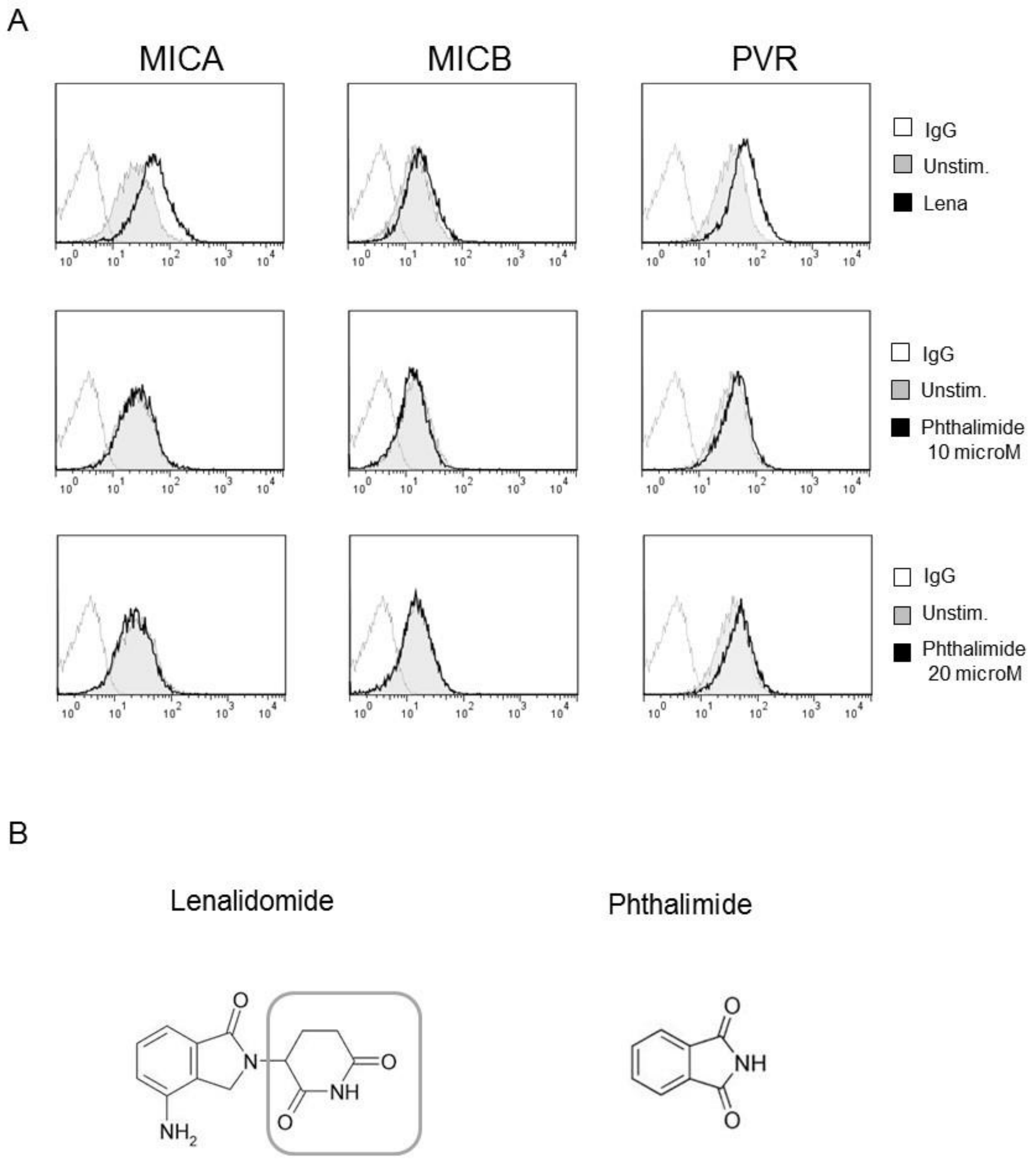

Fionda et al.
Supplemental Figure 5

Supplemental Figure 5 - **Effects of IMiDs on MICA and PVR/CD155 expression: molecular mechanisms.** A) MICA, MICB and PVR/CD155 surface expression were analyzed by flow cytometry on SKO-007(J3) cells treated with lenalidomide (Lena) (10 µM) or phthalimide (10 µM and 20 µM) for 72h. The grey colored histograms represent basal expression of the indicated ligand, while thick black histograms represent the expression after treatment with the indicated drug. Data are representative of one out of four independent experiments. B) Schematic structure of lenalidomide and phthalimide. The glutarimide moiety of lenalidomide is encircled.

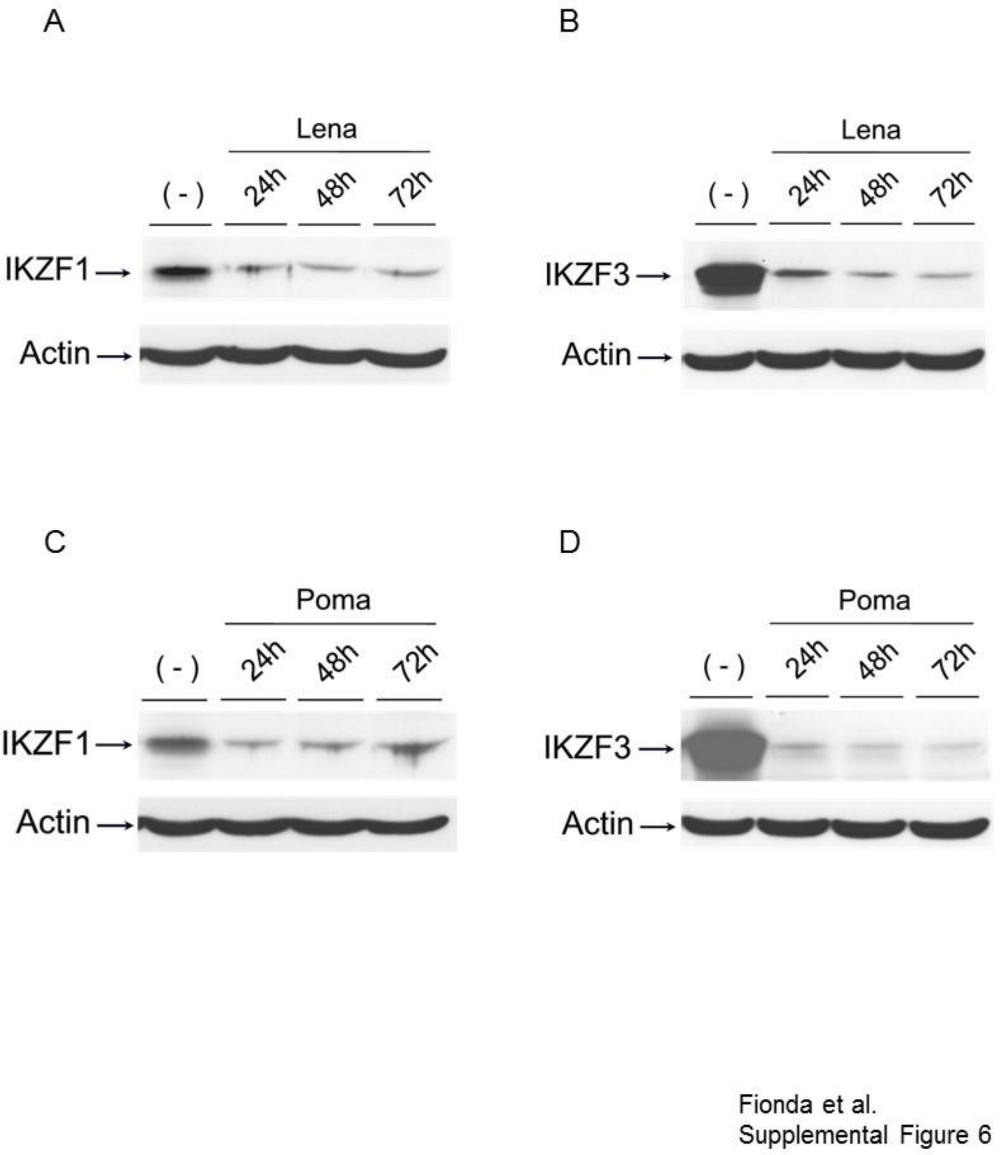

Supplemental Figure 6 - **IMiDs-mediated degradation of IKZF1 and IKZF3.** Immunoblotting analysis for IKZF1 or IKZF3 and Actin of total cellular proteins obtained from SKO-007(J3) cells treated with lenalidomide (Lena) (A,B) or pomalidomide (Poma) (C,D).

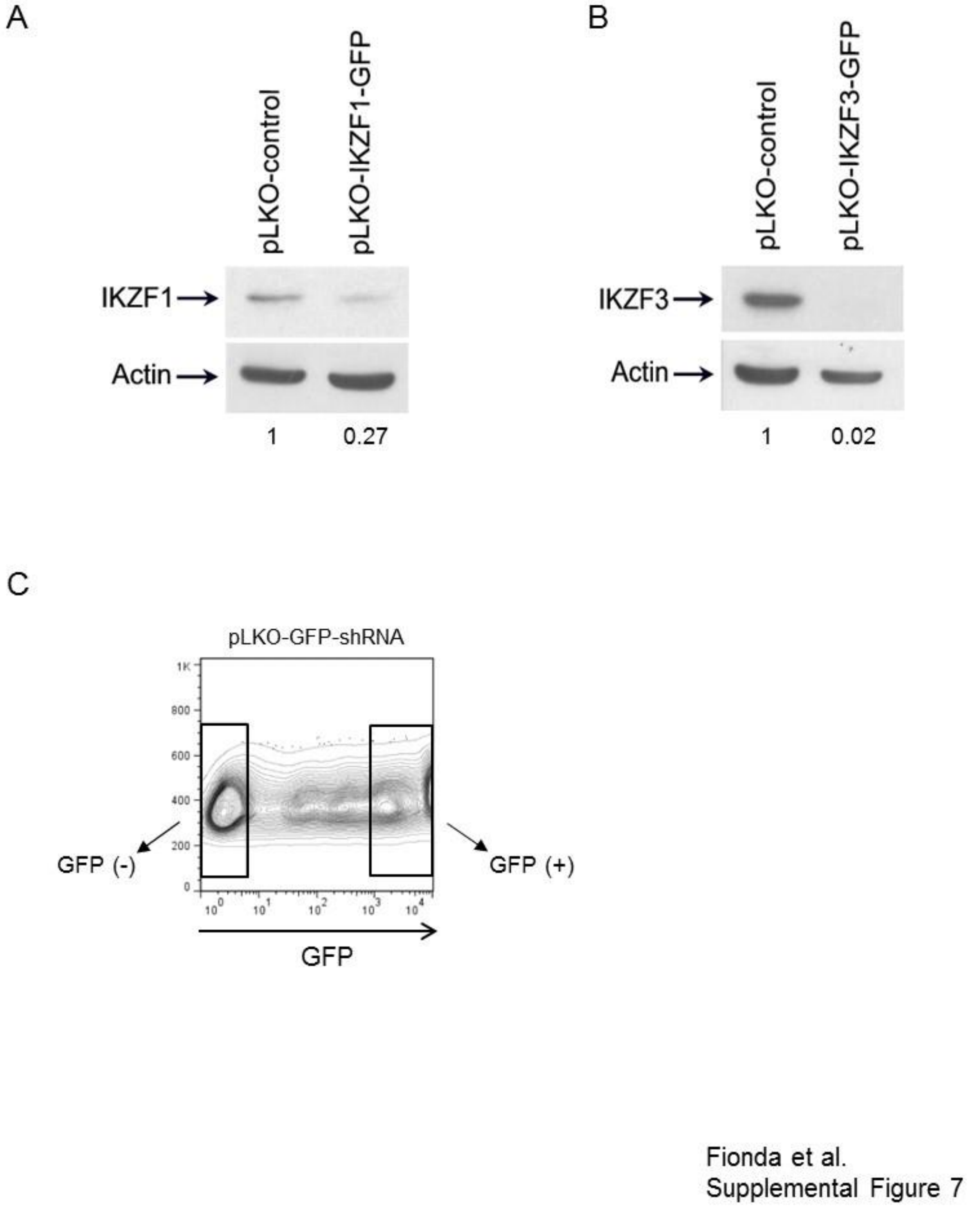

Supplemental Figure 7 - **Expression of IKZF1 or IKZF3 in SKO-007(J3) cells infected with lentiviral vectors expressing specific shRNAs.** Immunoblotting analysis for IKZF1 or IKZF3 and Actin of total cellular proteins obtained from SKO-007(J3) cells infected with lentivirus expressing IKZF1 (A) or IKZF3 shRNAs (B). The relative IKZF1 or IKZF3 amount was calculated by densitometry, normalizing to the level of Actin and expressed as fold change respect to the pLKO-mission control (arbitrarily set to 1). Data are representative of one out of three independent experiments. C) Gating strategy of pLKO-shRNA-transduced (GFP^+) and pLKO-shRNA-non-transduced (GFP^-) cells.

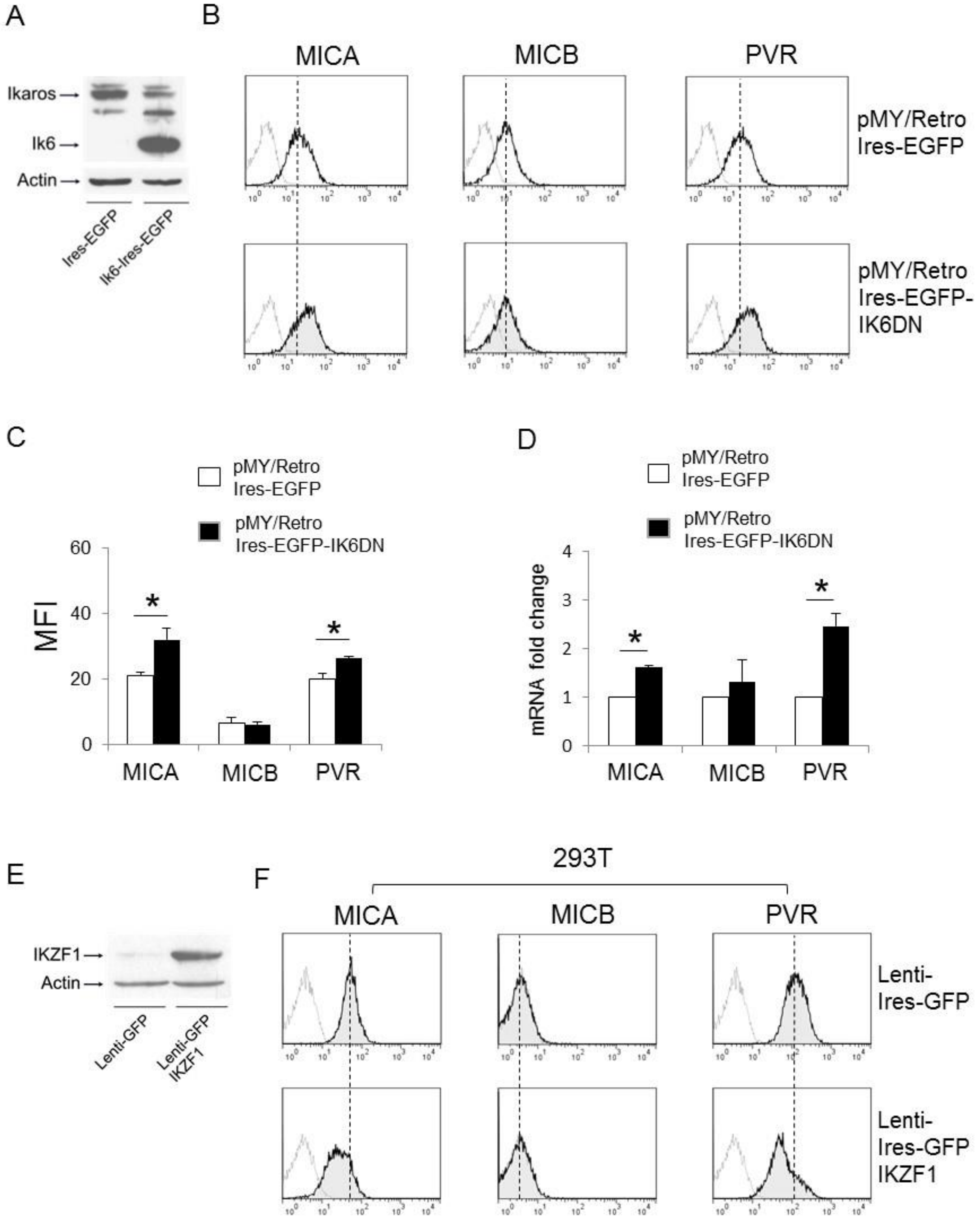

Fionda et al. Supplemental Figure 8

Supplemental Figure 8 - **IK6-DN overexpression enhances MICA and PVR/CD155 surface levels on SKO-007(J3) cells.** A) Immunoblotting analysis for IKZF1 and Actin of total cellular proteins from SKO-007(J3) cells infected with the retrovirus pMY/Retro-Ires-EGFP or MY/Retro-Ires-EGFP-IK6DN. The proteins transferred to nitrocellulose membranes were stained with Ponceau to verify that similar amounts of proteins had been loaded in each lane. Data are representative of one out of four independent experiments. B) MICA, MICB and PVR/CD155 surface expression were analyzed by flow cytometry on SKO-007(J3) cells transduced with a control retrovirus expressing EGFP (pMY/Retro-Ires-EGFP) or a dominant negative IKZF1 isoform, IK6 (MY/Retro-Ires-EGFP-IK6DN) as described in Supplemental materials and methods. Data are representative of one out of four independent experiments. The thick black histograms represent the expression of the indicated ligand in pMY/Retro-Ires-EGFP infected cells, while thick grey colored histograms represent the expression in pMY/Retro-Ires-EGFP-IK6DN infected cells. C) The MFI of MICA, MICB and PVR/CD155 were calculated based on four independent experiments and evaluated by paired Student *t* test (**P*< 0.05). For each treatment, histograms represent the MFI of specific mAb - MFI of isotype control. D) Real Time PCR analysis of total mRNA obtained from GFP^+ sorted SKO-007(J3) cells infected with pMY/Retro-Ires-EGFP or pMY/Retro-Ires-EGFP-IK6DN. Data, expressed as fold change units, were normalized with GAPDH and referred to the pMY/Retro-Ires-EGFP infected cells considered as calibrator and represent the mean of 3 experiments (**P*< 0.05). E) IKZF1 and Actin expression in 293T cells was analyzed by Western Blotting. The proteins transferred to nitrocellulose membranes were stained with Ponceau to verify that similar amounts of proteins had been loaded in each lane. Data are representative of one out of three independent experiments. F) MICA, MICB and PVR/CD155 surface expression were analyzed by flow cytometry on 293T cells infected with lentiviral vectors encoding GFP or GFP and IKZF1. Data are representative of one out of three independent experiments. The grey colored histograms represent the expression of the indicated ligand in GFP^+ infected cells.

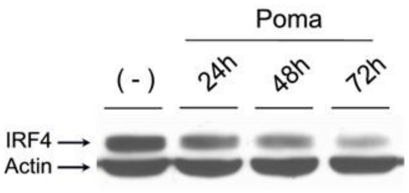

Fionda et al. Supplemental Figure 9

Supplemental Figure 9 - **IRF4 downregulation in SKO-007(J3) cells exposed to pomalidomide.** Lysates of SKO-007(J3) cells untreated or treated with pomalidomide (Poma) for 24h, 48h and 72h were subjected to Western Blotting using anti-IRF4 and Actin antibodies. The proteins transferred to nitrocellulose membranes were stained with Ponceau to verify that similar amounts of proteins had been loaded in each lane. Data are representative of one out of three independent experiments.
